# Angularly Resolved Tip‐Enhanced Raman Spectroscopy

**DOI:** 10.1002/anie.202506152

**Published:** 2025-07-20

**Authors:** Felix Schneider, Tim Parker, Liangxuan Wang, Michel Rebmann, Yang Zhao, Eric Juriatti, Heiko Peisert, Alfred J. Meixner, Johannes Gierschner, Lingyan Meng, Dai Zhang

**Affiliations:** ^1^ Institute of Physical and Theoretical Chemistry Eberhard Karls University of Tübingen Auf der Morgenstelle 15 72076 Tübingen Germany; ^2^ School of Physics and Physical Engineering Qufu Normal University P.R. China; ^3^ Madrid Institute for Advanced Studies IMDEA Nanoscience C/Faraday 9, Ciudad Universitaria de Cantoblanco Madrid 28049 Spain

**Keywords:** Angular‐resolved emission, Antenna directivity, Back focal plane imaging, Energy momentum spectroscopy, Tip‐enhanced Raman spectroscopy

## Abstract

Despite intensive research in tip‐enhanced Raman spectroscopy (TERS), the angular distribution of Raman scattering in the TERS gap remains experimentally unreported leaving its relevance to the TERS signal formation to be seldomly discussed. Here, we investigate the angular distribution of the tip‐enhanced Raman signal in the Fourier plane using a model system composed of flat‐lying cobalt (II) hexadecafluoro‐phthalocyanine (CoPcF_16_) molecules physically adsorbed on a smooth gold surface. Both in‐plane and out‐of‐plane vibrational modes are observed, where the out‐of‐plane Raman modes at about 678 and 740 cm^−1^ have different angular intensity distributions than those of in‐plane Raman modes at 1309 and 1373 cm^−1^. We interpret the angular spectrum of the TERS signal considering the molecular vibrational modes computed with density functional theory (DFT) for the free and gold‐deposited molecule, and the directed Raman scattering by the gap‐mode predicted by finite‐difference time‐domain (FDTD) simulations. We contend that the TERS gap directs the Raman vibrational modes differently, leading to distinct angularly distributed Raman scattering intensities. These findings emphasize the nonnegligible role of the TERS detection scheme in understanding spectral features, such as the relative peak intensity ratio variations for studying molecular orientations, or for monitoring chemical reactions.

## Graphical abstract

We find that the tip‐enhanced Raman signal (TERS) is angularly distributed and vibrational mode specific. The radiation pattern in the back focal plane (BFP) of vertical or parallel Raman dipoles is strongly influenced by the near‐field in the TERS nanogap, while the emission is guided into the far‐field with high directionality by the tip‐sample antenna; however, with significant differences in the emission angle, intensity, and the divergency.

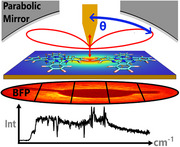

## Introduction

Raman spectroscopy is routinely used to analyze chemical structures of organic molecules. Generally, the optical resolution is limited by diffraction to roughly half a wavelength, which is much larger than the molecular dimension. One of the most prominent techniques to overcome this bottleneck is tip‐enhanced Raman spectroscopy (TERS), which was introduced independently by Stöckle et al.^[^ ^1^ ^]^ and Hayazawa et al.^[^ ^2^ ^]^ in 2000. Excited by a focused laser beam, a sharp metallic tip works as an optical antenna when it is brought near the sample surface. It enhances the incident optical field in the tip‐sample gap by several orders of magnitude and simultaneously guides the emission of photons from the gap into the far field.^[^ ^3^, ^4^ ^]^ In 2013 sub‐nanometer resolved single‐molecule Raman mapping (STM‐TERS, ∼80 K, 1.3 · 10^−8^ Pa) was demonstrated for the first time,^[^ ^5^ ^]^ pushing the spatial resolution with chemical identification down to ∼5 Å. In 2019, two further milestones were independently achieved.^[^ ^6^, ^7^ ^]^ Both groups demonstrated the unprecedent spatial resolution at the single‐chemical‐bond level. Soon afterwards, the spatially and spectrally resolved photoluminescence (PL) imaging of a single phthalocyanine molecule with an optical resolution down to about 8 Å was demonstrated.^[^ ^8^ ^]^ These achievements were well‐documented in a recent review article.^[^ ^9^ ^]^ The ability for such Å‐resolved spatial resolution to determine the chemical structure of single molecules arouse intense interests in the fields of chemistry, physics, materials, biology, and stimulated research to explore the super‐resolution mechanisms, to interpret the experimental data, and to mature the technique for wider applications.

So far, the state‐of‐the‐art TERS studies that address the signal enhancement mechanism from the narrow gap focus by discussing the spectral intensity and energy. Notably, a quantitative understanding of the near‐field enhancement based on intensity data needs to consider the angular radiation pattern of the sample emitters, the antenna directivity, as well as the angular dependent collection efficiency of the microscope setup.^[^ ^10^ ^]^ Generally, antenna‐enhanced emission is discussed in terms of the radiation efficiency η, the antenna directivity, and the antenna gain *G*. To quantify the capability of an antenna to radiate power preferentially into a certain direction, the antenna directivity is used: $D\;\left(\theta ,\phi \right)=\frac{p\;(\theta ,\phi )}{P_{r}/4\pi }\;$, which is defined as the ratio of the intensity *p* (θ, ϕ) radiated per unit solid angle to the emitted power $P_{r}=\int p\;\left(\theta ,\phi \right)\sin\theta d\phi d\theta \;$, where the angle θ is measured from the direction of the antenna and ϕ is the azimuthal angle. An equally important parameter is the antenna gain *G* (θ, ϕ), which is defined as the ratio of *p* (θ, ϕ) to the total input power *P_r_* + *P_nr_*, where *P_r_* is the radiative power and *P_nr_* is the ohmic loss of the antenna. The gain *G* (θ, ϕ) and directivity *D*(θ, ϕ) are related by the radiation efficiency of the antenna η as: *G* $\left(\theta ,\phi \right)=\frac{p\;(\theta ,\phi )}{(P_{r}\;+\;P_{nr})/4\pi }=\eta D\left(\theta ,\phi \right)$.^[^ ^4^ ^]^ Evidently, the angular distribution of the radiation intensity *p* (θ, ϕ) from the TERS gap is the key to understanding how the molecule interacts with the gap‐plasmon, and how optical signals are coupled out from the (sub‐)nanometer antenna‐gap forming certain TERS spectral features.

Although it has been theoretically predicted that the angular radiation intensity is highly sensitive to the gap configuration and the tip geometry,^[^ ^11^, ^12^, ^13^ ^]^ there has been little systematic experimental work done in this regard. The absence of relevant experimental studies can be related to the setup configurations conventionally used in the field of tip‐enhanced optical microscopy, where only a fraction of the emitted signals is collected or simulated from one side of the tip at 60–70 degrees versus the optical axis above the sample.^[^ ^8^, ^14^ ^]^ Another common TERS configuration uses an objective lens installed below the sample to collect the scattered radiation. In this case, the optical signal needs to be collected through the substrate. Therefore, the modification of the emission pattern through the dielectric substrate and the internal reflection at the molecule‐substrate interface need to be considered.^[^ ^15^, ^16^ ^]^ Hence, it is highly challenging to observe a broad angular range of the intrinsic emission pattern of the TERS signal.

**Figure 1:**
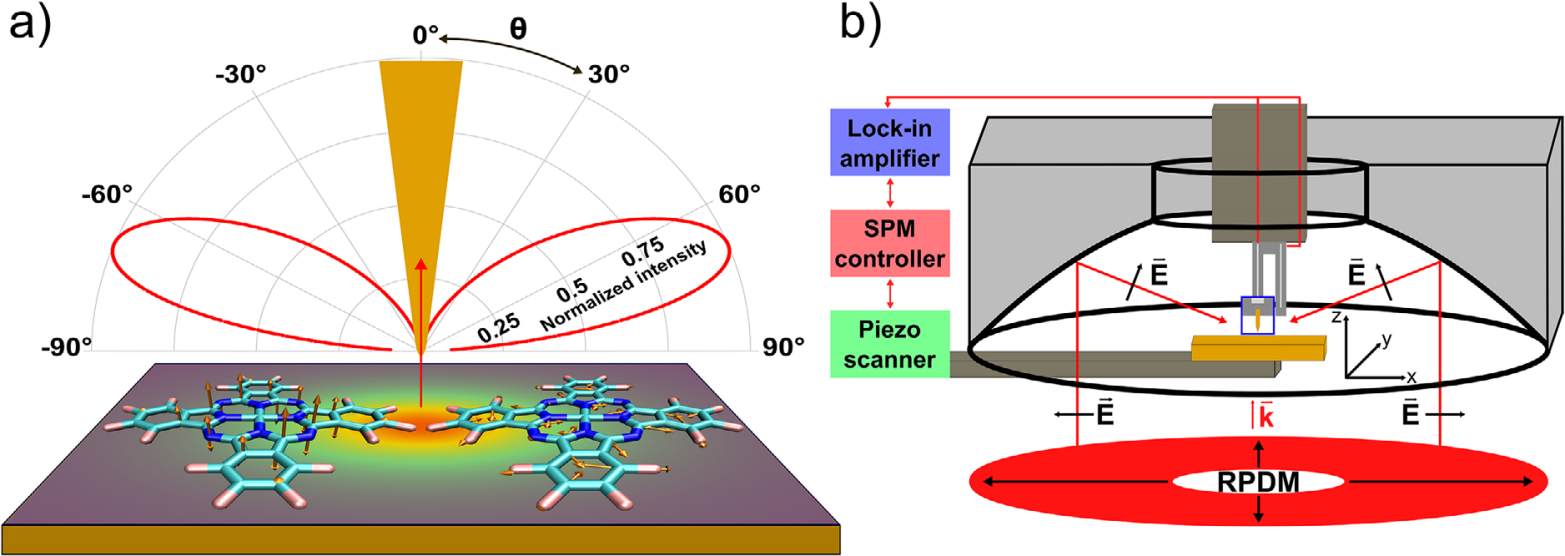
Experimental setting. a) Scheme of the angular resolved radiation intensity from flat‐lying CoPcF_16_ molecules in a TERS gap with the in‐plane and out‐of‐plane Raman dipoles. b) Top‐illumination TERS using a parabolic mirror to focus the RPDM beam onto the tip‐sample gap.

In this work, we explore the angular distribution of the TERS signals of CoPcF_16_ molecules that are adsorbed on an ultra‐smooth gold surface collected by a parabolic mirror as outlined in Figure 1. Due to the large numerical aperture (0.9986 in air) of the parabolic mirror positioned above the gold substrate, the Raman signals scattered from the tip‐sample gap can be collected from about 27 to 87 degrees symmetrically around the optical axis. This configuration largely satisfies the requirements of correlating the intensity and angular information in a TERS measurement. As probe molecule, we choose fluorinated cobalt (II) phthalocyanine molecules because of their high photochemical stability, their rigid molecular structure, and their tendency to form layers with preferred flat lying molecular orientation. Such a molecular orientation was shown for several transition metal phthalocyanine molecules, including CoPcF_16_, on single crystalline metal surfaces, and for low coverages also on comparably rough gold substrates.^[^ ^17^, ^18^ ^]^ Due to these benefits, many of the milestone sub‐molecular TERS studies used similar types of organic molecules.^[^ ^5^, ^6^, ^7^, ^8^ ^]^ Therefore, the outcome of our study on CoPcF_16_ is highly relevant to the frontiers of TERS research. To record the angular distribution of scattered optical signal, we implement a back‐focal plane detection scheme in the Fourier plane of our microscope. To distinguish Raman scattering from the PL intensity, we perform energy momentum TERS spectroscopy by spatially selecting the optical signals from specific angular ranges and detect them spectrally using a CCD‐coupled spectrometer.

We observe the gap‐distance dependent TERS enhancement from flat‐lying CoPcF_16_ molecules on ultra‐smooth gold surfaces in the Stokes, anti‐Stokes, and the overtone regions that are different from the recently reported “TERS quenching”.^[^ ^19^ ^]^ We demonstrate that the TERS enhancement is strong enough to visualize the optical signal from the tip‐sample gap in sections of the Fourier plane and to spectrally analyze them using energy momentum spectroscopy. We collect strong Raman spectra from individual angular ranges and reveal differences in the angular resolved TERS intensities of individual vibrational modes. We analyze these differences considering the in‐plane or out‐of‐plane nature of the respective vibrational modes and the directed scattering by the tip‐sample configuration. We confirm that angular Raman scattering is greatly impacted by the gap‐plasmon mode in the near‐field, while the directionality is determined by the antenna. Our findings demonstrate the high relevance of the angular spectrum for the understanding of the TERS spectral features.

## Results and Discussion

### TERS of CoPcF_16_ Molecules on a Gold Surface

The impact of the excited gap‐plasmon mode on the Raman signals of the molecules is illustrated in Figure 1a. The angular distribution of the Raman dipole emission is affected by the local electric field in the nanometer gap in several ways. First, the total radiated power can be enhanced through the excitation of gap plasmon resonances. Second, the angle‐resolved radiation intensity *p*(θ, ϕ) of the Raman dipole can be altered by gap‐plasmon radiation behavior. These effects lead to the radiated power per solid angle being modified by the radiation enhancement. If the Raman dipole frequency coincides with a certain plasmonic resonance, the emission pattern of Raman scattering will be greatly modified by this plasmonic mode.

To study this process experimentally, the TERS configuration shown in Figure 1b is used. Detailed information about the TERS setup can be found in Supporting Information 1. An electrochemically etched gold tip is brought into proximity of the sample via a mechanically excited shear‐force tuning fork. Decreasing the tip‐sample distance is controlled by approaching the sample to the tip while monitoring the phase shift of the tuning fork oscillation. In our experiments, for the TERS gap consisting of a gold tip and a gold surface, typically for a phase shift of 4.5 degrees, the tip‐sample gap is about 4 nm, henceforth referred to as “large gap”. Increasing the phase shift to 15 degrees decreases the gap distance to about 2 nm, henceforth referred to as “small gap”.^[^ ^20^ ^]^ To excite the gap‐plasmon mode efficiently along the optical axis a radially polarized donut mode (RPDM) with a wavelength of 633 nm is used. For comparison, an azimuthally polarized donut mode (APDM) possessing exclusive x‐y field intensity |*E*_x,y_|^2^ is also used. Detailed information about the field distributions of RPDM and APDM can be found in Supporting Information 1. A CoPcF_16_ film of about two layers thick (0.7 nm) is evaporated onto an ultra‐smooth gold substrate without a spacer layer in between as a sample, see Supporting Information 2.

**Figure 2:**
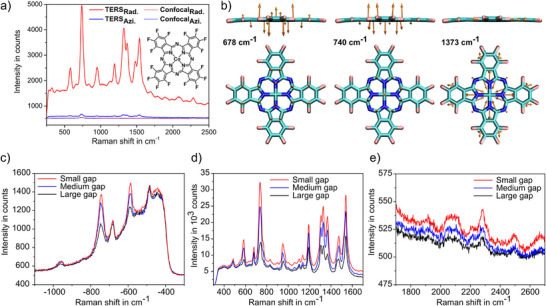
TERS spectra and calculated Raman vibrational motions. The excitation wavelength is *λ* = 633 nm, the acquisition time for the Stokes spectrum is 30 s, and for the anti‐Stokes spectrum is 180 s. a) Raman spectra without tip engagement (dotted lines) and with tip engagement (solid lines) of CoPcF_16_ on a smooth gold film with different excitation polarizations. Inset: Chemical structure of CoPcF_16_. b) Nuclear displacements of vibrational modes calculated for $\tilde{\nu }$ = 678, 740 (out‐of‐plane), and 1373 cm^−1^ (in‐plane) using DFT. c)–e) Anti‐Stokes‐, Stokes‐, and overtone Raman scattering for small, medium, and large gap distance, respectively.

Experimentally acquired TERS spectra are shown in Figure 2a. Using RPDM (red solid line) leads to significant enhancement as compared to the spectrum taken without a tip (red dotted line). This complies with the strong vertical gap‐mode that is excited by the dominant *E*_z_ component in the focus of a RPDM beam. The TERS spectrum taken with an APDM (blue solid line) is also shown. As compared to the case without tip (blue dotted line), the APDM‐TERS spectrum also shows Raman enhancement, though it is moderate compared to the RPDM‐TERS. These results further confirm the effective excitation of the vertical plasmonic gap mode that is essential for the electromagnetic (EM) enhancement of the TERS signal. To analyze the individual Raman vibrational modes, we have calculated the resonant‐ Raman active modes using (time‐dependent) density functional theory ((TD‐)DFT) for CoPcF_16_ on a gold substrate considering the adsorption geometry and the gold‐tip gold‐substrate configuration (see Supporting Information 3). Most of the prominent Raman‐active peaks correspond to in‐plane vibrational modes, such as 967, 1194, 1307, 1335, 1373, 1474, and 1540 cm^−1^. However, there are two peaks associated with out‐of‐plane modes at 678 and 740 cm^−1^, which can be assigned to vibrations of C‐ and N‐atoms in the macrocycle structure (see Figure 2b). The vibrational geometry will be essential to elucidate the angularly resolved Raman scattering discussed later.

We note that different from the recent work reporting the TERS quenching from ZnPc molecules,^[^ ^19^ ^]^ we observe strong TERS enhancements. There are a few factors that could contribute to these different phenomena. First, in our study a CoPcF_16_ film with a nominal thickness of 0.7 nm is used. In the case of homogeneously distributed molecules over the gold surface, this nominal thickness accounts for two layers of CoPcF_16_ molecules that are flat‐lying on the substrate. This is different from the single molecule case reported by Yang et al.^[^ ^19^ ^]^ Secondly, the parabolic mirror collects optical signals around the TERS gap over a much larger angle range than it is for the 60 degrees TERS detection configurations. This different detection scheme needs to be considered, which will be detailed in this paper. Finally, the tip used in our study is not specially designed for having atomic protrusions at the tip apex. Combined with the larger tip‐sample distances in the nm regime, the field confinement and polarization are different from the aforementioned work.

Further, we observe that the Stokes and anti‐Stokes peaks as well as the overtones all increase as the gap‐distance decreases, confirming the essential role of EM enhancement in our study (see Figure 2c–e). Notably, our previous work has shown that charge transfer occurs between CoPcF_16_ and the gold substrate, and this process is expected to be bidirectional, involving both the central metal atom and the macrocycle.^[^ ^18^ ^]^ Therefore, the contribution of chemical enhancement to the different Raman peaks cannot be excluded. A closer look at the individual peak ratios in the anti‐Stokes region reveals different peak ratios as compared to their corresponding Stokes lines. For example, $I_{-740\;cm^{-1}/-678\;cm^{-1}}$ is 3.4, and the $I_{740\;cm^{-1}/678\;cm^{-1}}$ is 7.8. We consider the selective Raman peak enhancement, e.g., the anti‐Stokes peak at −678 cm^−1^, to be related to its proximity to the gap plasmon resonance maximum. Previous findings in surface‐enhanced resonance Raman spectroscopy (SERRS) studies have shown that the peaks close to the plasmon resonance maxima are selectively enhanced.^[^ ^21^ ^]^ We then indirectly derive the gap plasmon resonance information from the spectral background of the TERS spectrum. The broad “continuum” TERS (and SERS) spectral background, though still under debate,^[^ ^22^, ^23^, ^24^ ^]^ has been shown to be closely related to the metal PL and is impacted by the plasmon resonance.^[^ ^20^, ^25^, ^26^, ^27^, ^28^, ^29^, ^30^, ^31^ ^]^

As shown in Figure 2d, the maximum of the spectral background is at 1.890 ± 0.005 eV at small gap distance, which blue shifts to 1.905 ± 0.007 eV at large distance, a phenomenon that is typical for the distance‐dependent gap‐plasmon mode (for details, see Supporting Information 4). In Figure 2c, the −678 cm^−1^ anti‐Stokes peak is closer to the spectral background maximum, hence is more enhanced by the gap‐plasmon mode than the −740 cm^−1^ one. Indeed, at the far‐end of the spectral background, the last anti‐Stokes Raman peak at −967 cm^−1^ is just visible, indicating the significantly decreased impact from the gap‐plasmon resonance. In other experiments, where the TERS spectral background is much broader than the one in Figure 2c, we can observe the anti‐Stokes peaks up to −1540 cm^−1^ (see Figure S6a).

### Simulated Directed Emission of a TERS Configuration

**Figure 3:**
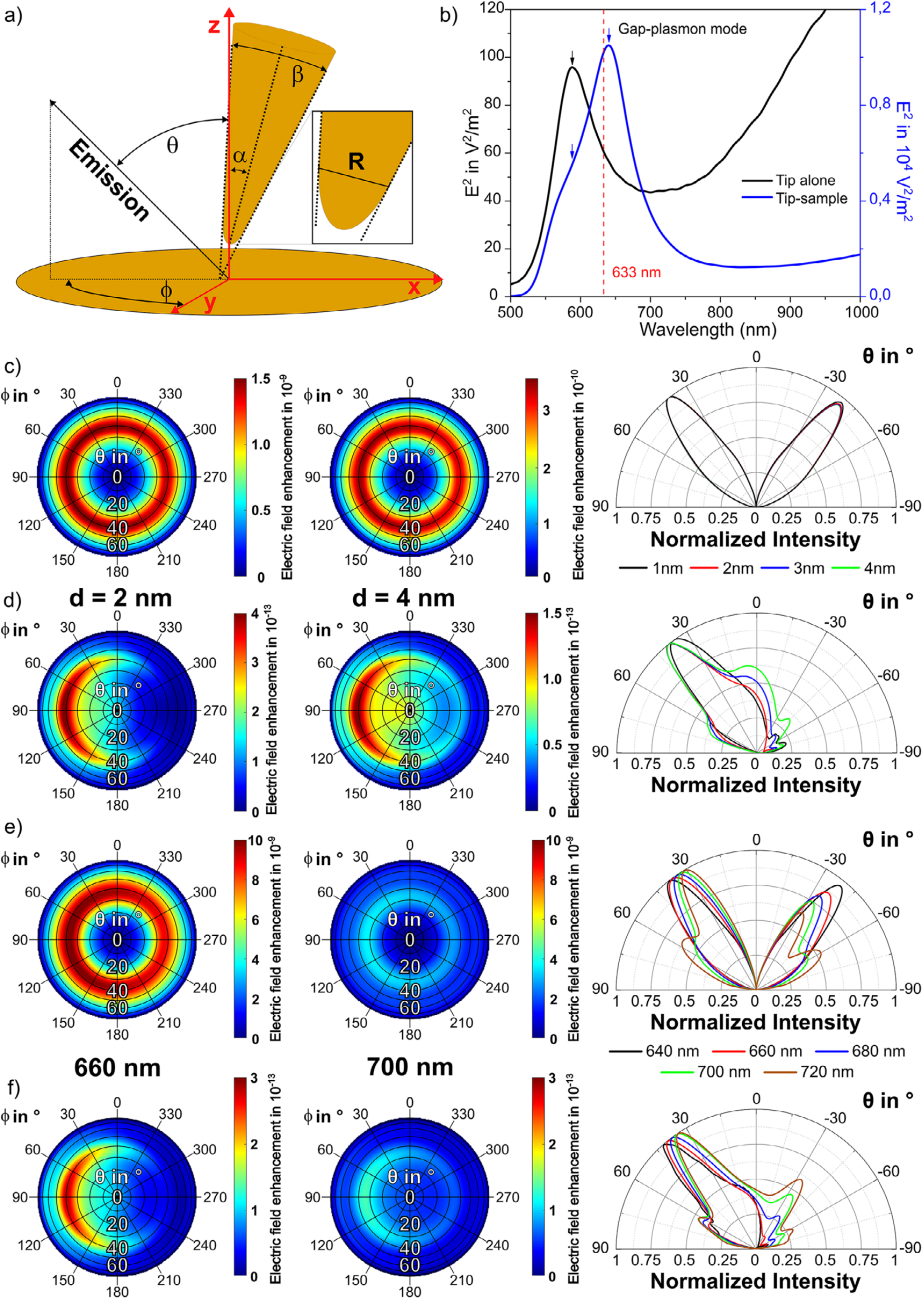
Calculated gap‐distance and wavelength‐dependent far‐field emission intensity |E^2^|. a) Definition of simulation parameters: *θ* is the polar angle versus the optical axis (z), and *φ* is the azimuthal angle in the x‐y‐sample plane. b) Simulated plasmon resonances for the tip alone, and the tip‐sample gap (*d* = 2 nm). c) and d) Radiation patterns for λ_*em*_= 640 nm at varying gap‐distances simulated for an emitter dipole c) vertical, and d) parallel to the sample plane, respectively. Right: Normalized polar plots in the x‐z‐plane for varying gap‐distances, sectioned from *φ* = 90° to *φ* = 270° in the respective polar plots. e) and f) Radiation patterns for different emission wavelengths (*λ* = 640, 660, 680, 700, 720 nm) calculated for a dipole e) vertical, and f) parallel to the sample plane in a 2 nm tip‐molecule gap, respectively. Right: x‐z‐sections of normalized emission intensity for varying wavelengths, sectioned from *φ* = 90° to *φ* = 270° in the respective polar plots.

We explore the angularly distributed optical signals in the far‐field using the finite‐difference time‐domain (FDTD) method (see Supporting information 5). The simulation model consists of a slightly tilted gold tip above a gold film that is covered with a CoPcF_16_ thin film with a thickness of 1 nm. The tip geometry is defined by the apex diameter (R), opening angle (β), and tilting angle with respect to the optical axis (i.e., the sample normal) (*α*) shown in Figure 3a. To comply with the experimental conditions and the real experimental tip shape observed in a scanning electron microscope image, α, β, and R of the gold tip are simulated as *α* = 3 °, *β* = 18 °, and *R* = 70 nm, respectively. The plasmon resonance of such a tip‐sample gap is calculated as 640 nm for a gap distance of 2 nm, which is blue shifted to 587 nm for the tip alone (Figure 3b).

We first calculate the far‐field emission of a dipole (either vertical or parallel to the sample plane) in a tip‐sample configuration. The results show that in both cases there is a highly directional emission pattern. In detail, the far‐field emission pattern for the vertical (to the sample surface) dipole emerges in a uniform ring (Figure 3c) that is nearly independent of the azimuthal angle (*φ*) but strongly depends on the polar angle (*θ*). In contrast, guided by the slightly tilted tip, the far‐field emission for the parallel (to the sample surface) dipole is shown in a crescent shape that depends on both *θ* and *φ*. The parallel dipole also couples to the gap mode due to the complex electromagnetic field in the tip‐film nanogap, in which the vertical and parallel components of the localized electric field exist simultaneously though with different strengths.^[^ ^32^, ^33^, ^34^ ^]^

We then compare the gap‐distance dependent far‐field emission intensity patterns for various *θ* angles in the x‐z‐plane of the polar plots shown in Figure 3c,d. Three characteristics can be found: (1) The vertical dipole shows two main symmetric lobes at θ = ± 39°. In contrast, for a parallel dipole (Figure 3d), most of the radiation concentrates in one main lobe emission pattern with the strongest signal at *θ* = 40° on the opposite side of the tip‐tilt angle (here at *φ* = 90°). For both cases, the maximum emission angle remains almost unchanged as decreasing the tip‐sample distance from 4 to 1 nm. Notably, for the parallel dipole the relative emission intensity ratio $I_{\theta =0^{\circ }}/I_{\theta =40^{\circ }}$ is increasing for larger tip‐sample distances, indicating the decreased directivity of the tip antenna. (2) The far‐field emission intensity for the vertical dipole is changing faster at varying gap distance than that for the parallel dipole. It indicates that the vertical dipole interacts with the tip with larger coupling strength *g_e_* (see Supporting Information 5) than the parallel dipole. Indeed, the far‐field emission intensity for the vertical dipole is approximately four orders of magnitudes larger than that for the parallel dipole at the same tip‐sample distance *d*. This is in good agreement with previous experimental results, where mostly the out‐of‐plane vibrational modes appeared in the TERS spectra.^[^ ^35^, ^36^ ^]^ (3) The emission pattern of the vertical dipole shows a smaller divergence angle (∼28°), which is defined as the full width at half maximum (FWHM) of the radiation lobe, than that of the parallel dipole (∼37°) at *d* = 2 nm. The divergence angle for the vertical dipole remains unchanged while that for the parallel dipole is increased from ∼37° to ∼59° as increasing the gap distance d from 2 to 4 nm. Hence, we conclude that the antenna directivity is stronger for a vertical dipole than for a parallel dipole. We continue the investigation by simulating the wavelength‐dependent directivity by the tip‐sample nanogap. As shown in Figure 3e,f, the closer the emission wavelength to the plasmon resonance, the higher the intensity and the smaller the divergence are.

We summarize the findings from the simulation results. The plasmonic near‐field distribution in the tip‐sample nanogap shapes the radiation pattern, and the tip‐sample configuration guides the far‐field emissions of vertical and parallel dipoles resulting in high directionality; however, with significant differences in the maximum emission angle, intensity, and the divergence. The directed emission is both gap‐distance and wavelength dependent. These distinct angularly distributed far‐field emission characteristics lead to different detection efficiencies for the radiation dipoles of varying orientations (see Supporting Information 6). Although the vertical dipole is coupled to the tip more efficiently, it should be noted that a side‐illumination TERS setup may have a higher signal collection efficiency for a parallel dipole compared to a vertical dipole due to its single lobe emission pattern. We assume a case that an aspherical lens with a NA of 0.51 positioned at an angle of 60° from the surface normal is used to detect the optical signal from a parallel dipole in a tip‐sample gap where the tip is slightly tilted (*α* ≈ 3°). If the lens is located at the single lobe direction, the detection efficiency would be 20.5%, while it is only 6.4% if the lens is located at the opposite side (see Figure S11). For a vertical dipole, the lens position is comparably less critical because of its more symmetric emission pattern, which leads to a detection efficiency between 14.9% and 12.6%. Thus, the angular radiation of different vibrational modes, and the position of the objective lens should be considered to interpret the TERS spectral features, particularly the relative peak ratios between in‐plane and out‐of‐plane vibrational modes.

### Experimental Angularly Resolved TERS Signal

**Figure 4:**
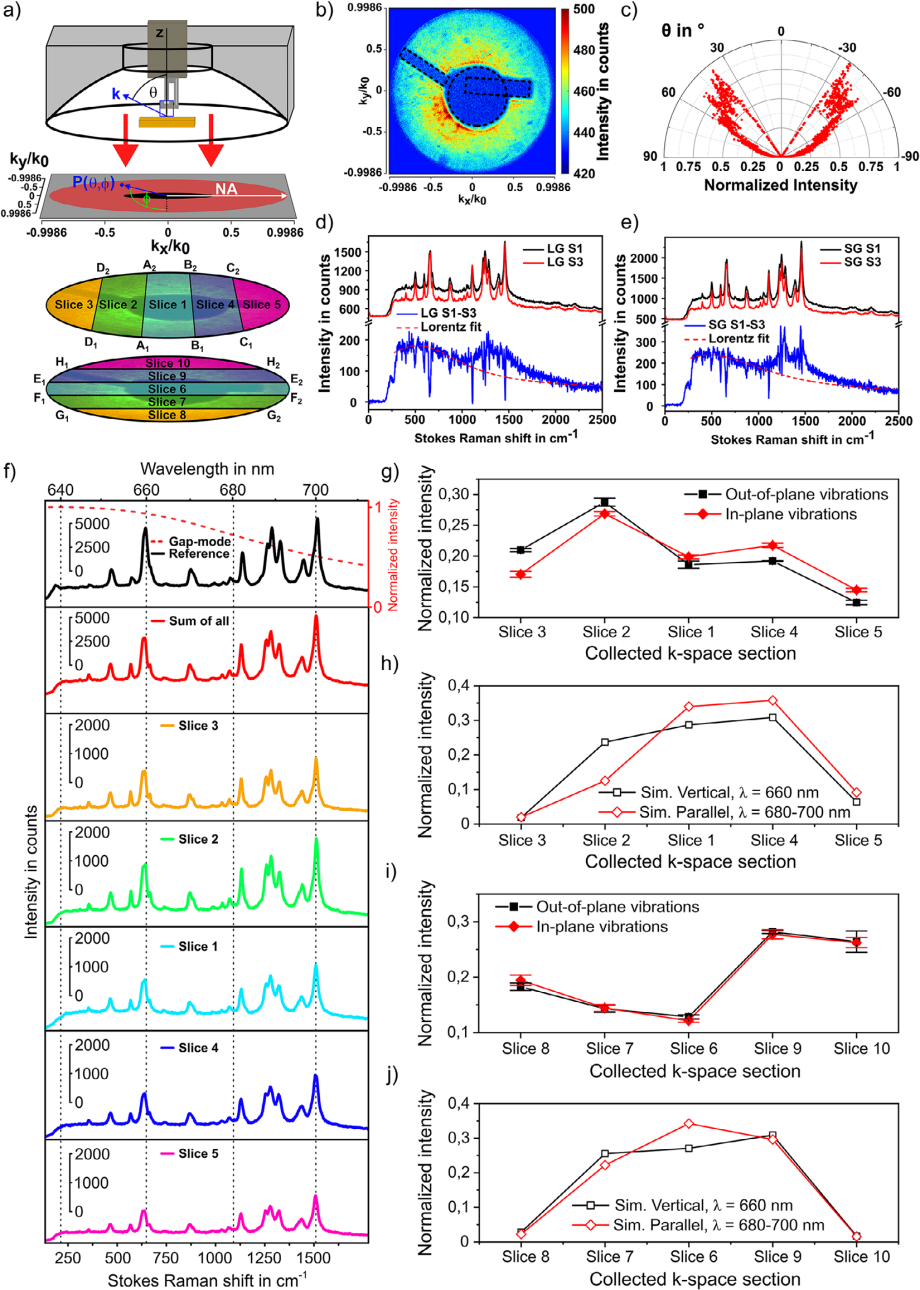
Angularly resolved TERS. The excitation wavelength is *λ* = 633 nm; the acquisition time is 90 s for all Raman spectra and 180 s for back‐focal plane images. a) Back‐focal plane and energy momentum TERS setup scheme. The collected *k‐*space image is limited by the numerical aperture of the parabolic mirror. The angular range marked as black cannot be detected due to the hole in the parabolic mirror. Spatial slicing is used to select TERS signal from a defined angular range. b) Back‐focal plane image recorded at a tip‐sample gap of about 2 nm. c) Radiation intensity at varying *θ* angle in a 2 nm tip‐sample gap. d) and e) Raman spectra collected in Slice 1 (S1) and Slice 3 (S3) at large gap (LG) and small gap (SG), respectively. The rest of the subtracted spectra is shown in blue. The red dashed line indicates the spectral background fit. f) TERS spectra collected in five angular ranges, and the reference TERS without angular selection. The red dash line indicates the gap plasmon resonance calculated in Figure  3b in the spectral range of 640 and 710 nm. g) Experimental and h) simulated far‐field emission intensity from Slice 1 to Slice 5 for dipoles vertical or parallel to the sample plane, respectively. i) and j) The same as g) and h) but for Slices 6 to 10.

We implement the back‐focal plane (BFP) imaging technique^[^ ^37^, ^38^, ^39^ ^]^ to the TERS setup (see Supporting Information 7) to visualize the radiation pattern experimentally. Shown in Figure 4a, the radiation intensity *p* (θ, ϕ) of a dipole in a TERS gap is projected to the BFP indicated as a red disk. The BFP pattern is scaled by the normalized wavevector components as *k_x_*/*k*_0_ and *k_y_*/*k*_0_, where $k_{0}=\frac{2\pi }{\lambda }\;$. The dimension of the BFP image is determined by the NA of the parabolic mirror that allows the maximum collection angles of *θ* = 87 °and *φ* = 360 °. We note that in our experiment, only the polar angle range from *θ* = 27° to 87° is collected. The angular information (*θ*, *φ*) of the inspected signal is derived from the image considering the parabola equation of the parabolic mirror. Figure 4b shows an experimental BFP image taken from a 2 nm TERS gap. Intense signals are distributed in the range of *θ* = 35 to 55 °, see Figure 4c). The area with no intensity is due to the shadowing of the sample holder and one prong of the tuning fork (see dashed area).

We further implemented the energy momentum spectroscopy technique^[^ ^40^, ^41^, ^42^, ^43^ ^]^ to the TERS setup to distinguish the angularly distributed sharp TERS peaks from the broad PL spectral background. This is achieved by spatially slicing optical signals of certain angles (θ, ϕ) as shown in Figure 4a, and spectrally dispersing them with a CCD‐coupled spectrometer (for more details, see Supporting Information 7). The Slices 1–5 are sectioned parallel to *k_y_*, while Slices 6–10 are sectioned parallel to *k_x_*. Figure 4d compares the energy momentum TERS spectra that are taken from two different angular ranges shown as Slice 1 and Slice 3 at the tip‐sample distance of 4 nm. Here, Slice 1 covers all points in between the two lines from A_1_ to A_2_ and B_1_ to B_2_ in Figure 4a. Similarly, Slice 3 contains all points between the line from D_1_ to D_2_ and the left edge of the aperture in Figure 4a. The precise angular information for all sections is summarized in Table S2. When subtracting the TERS spectrum taken from Slice 1 by that from Slice 3, the following features can be clearly seen in Figure 4d: (1) Raman peaks in the spectral range of 473–1129 cm^−1^ appear as “dips” in the subtracted spectrum indicating their lower intensities in the angular range of Slice 1; (2) Raman peaks in the spectral range 1184–1540 cm^−1^ still show negative intensities, but the PL background turns positive. These phenomena are more significant for the smaller tip‐sample distance of 2 nm in Figure 4e, where the subtracted Raman peaks in the 1184–1540 cm^−1^ range turn positive.

We analyze the energy momentum TERS spectra by deriving the angularly distributed Raman intensities for all prominent Raman modes and compare them with the simulated results in Figure 3. Figure 4f shows a series of TERS spectra taken from different angle ranges. The top‐most spectrum is a normal TERS spectrum without angular selection. The second spectrum from the top is the sum of all energy momentum TERS spectra taken from Slices 1 to 5. These two spectra have similar peak intensity ratios. However, the intensity of the individual Raman modes in the spectra taken from different angle ranges varies in distribution. To manifest these variations, we integrate the intensity of a certain vibrational mode for each angle range and then normalize it by the total intensity of this vibrational mode in all angular slices. Taking the Raman peak at 678 cm^−1^ (out‐of‐plane vibrational mode) as an example, the normalized intensity $I_{norm.\;678\;cm^{-1}}$ is derived as $\frac{I_{678\;cm^{-1}\;in\;Slice\;1}}{I_{678\;cm^{-1}\;in\;Slice\;1\;to\;5}}$. The normalized intensity $I_{norm.\;740\;cm^{-1}}$ of the other out‐of‐plane vibrational mode is derived in the same way. Afterwards, the value of $\frac{I_{norm.\;678\;cm^{-1}}+I_{norm.\;740\;cm^{-1}}}{2}$ is used to plot the y‐axis and the sequence of the spatial section is shown as the x‐axis in Figure 4g to illustrate the angularly distributed Raman intensity of all out‐of‐plane vibrational modes. The same procedure is carried out for all in‐plane vibrational modes (967, 1309, 1331, 1373, 1469, and 1540 cm^−1^), giving rise to the red line in Figure 4g. The simulated far‐field emission from a vertical or parallel dipole, in analogue to the out‐of‐plane or in‐plane vibrational modes, was processed in the same manner, and the result is shown in Figure 4h. Following the same procedure, Slices 6 to 10 were processed, see Figure 4i,j.

We first compare the evolving tendencies of out‐of‐plane modes with those of the in‐plane modes at different angular ranges. We observe that out‐of‐plane and in‐plane vibrational modes have different intensity distribution in the inspected ten angular ranges. For example, in the angular range of Slice 2 (see Figure 4g), the normalized intensity of the out‐of‐plane Raman modes is larger than that of the in‐plane vibrational modes, which inverts for Slices 1, 4, and 5. This tendency is in line with the simulated results (see Figure 4h). It is important to realize from inspection of Figure 4g that the average intensity of the out‐of‐plane vibrational mode in Slice 2 is approximately 231% higher than in Slice 5. We observe distinct angular distributions for the Raman intensities of out‐of‐plane and in‐plane vibrational modes. For example, in Slice 3 of Figure 4g, the average out‐of‐plane vibrational mode intensity is approximately 24% higher than that of the in‐plane vibrational modes. For the Slices 4 and 5, the average in‐plane vibrational mode intensity is higher by 14% and 17%, respectively. For Slices 6 to 10, the normalized intensities of the out‐of‐plane modes are similar to the in‐plane modes, as shown both in the experimental results (see Figure 4i) and simulations (see Figure 4j). In sum, in Figure 4 we experimentally demonstrate the angular distribution of TERS signals. We performed the same experiments and analysis for a medium and large tip‐sample distance, the results are shown in Figure S13 in the Supporting Information. The impact of the tip‐sample distance on the angular distribution of the Raman scattering signal is weak in the studied gap distance range, which agrees with the FDTD simulation results shown in Figure 3c,d.

We note that although the changing tendencies of the experimental results go along with the simulated ones in most of the cases, for some angle ranges, such as Slice 1 and 6, the agreement between experiment and simulation is less satisfactory. To understand the discrepancies, the following considerations need to be made: (1) As seen in the BFP images, part of the sample holder and one prong of the tuning fork block and/or scatter the optical signal, which modifies the observed radiation patterns of the Raman vibrational modes. (2) CoPcF_16_ thin film has been used instead of a single molecule. Slight variations in molecular orientations cannot be excluded, which makes the comparison between the simulation and the experiment less consistent. (3) The realistic tip geometry in the tip‐sample gap is approximated as the true geometry regarding the sub‐nanometre structure of the tip apex cannot be imaged. It has been well‐accepted that atomic protrusions play a significant role in the TERS signal generation^[^ ^44^, ^45^ ^]^ though the stability and thus the effect of such protrusions under ambient condition is still unclear.^[^ ^46^, ^47^ ^]^ In our simulation, no such kind of atomic features have been considered, which could potentially bring further inconsistencies between the simulation and the experiments.

We interpret the observations above by considering vibrational mode geometry, and wavelength‐dependent directivity. As shown in Figure 3e,f, the closer the emission wavelength is to the nanogap resonance, the stronger the intensity, and the smaller the divergence are. In Figure 4d,e, the spectral background maximum locates at 651 and 656 nm, which are close to the simulated gap plasmon resonances in Figure 3b. The two out‐of‐plane Raman vibrational modes (678 cm^−1^, 740 cm^−1^) scatter at approximately 660 nm, which are closer to the gap plasmon resonance than all in‐plane Raman modes (967, 1309, 1331, 1373, 1469, and 1540 cm^−1^) which scatter between 680 and 700 nm. In turn, the radiation patterns of the out‐of‐plane Raman vibrational modes (678 cm^−1^, 740 cm^−1^) are significantly directed by the tip antenna, giving rise to the stronger scattered far‐field intensity in the larger polar angles such as in Slice 3, rather than in Slice 1. Thus, these peaks appear to be “dips” in the subtracted spectra in Figure 4d,e. Comparably, the wavelength of the scattered photons in the spectral range 680–700 nm is less effectively overlapped with the gap mode resonance. Hence, in this spectral range the gap mode impacts on the angular distribution of the TERS signal less significantly. Here, intensity features of the few in‐plane Raman modes are more complex, where both “dips” and “peaks” are visible. Interestingly, the peak widths are slightly increased for some peaks (1309, 1331, 1373, and 1540 cm^−1^), giving rise to “peaks” in the subtracted spectra. Further studies need to be performed to understand these details.

## Conclusion

In summary, the parabolic mirror assisted‐energy momentum TERS results clearly reveal the vibrational mode‐specific angular distribution of Raman intensity. The radiation pattern of vertical or parallel dipoles is strongly influenced by the near‐field in the tip‐sample nanogap, while the emission is guided into the far‐field with high directionality by the tip‐sample antenna; however, with significant differences in the maximum emission angle, intensity, and divergency. They demonstrate that the TERS signal is highly angle‐dependent, which could induce the large variations in the TERS spectral features among results from different TERS setups (side‐illumination/collection, parabolic top illumination/collection, and bottom illumination/collection), or from the same setup but with different tips.

## Supporting Information

The authors have cited additional references within the Supporting Information.^[^ ^7^, ^29^, ^48^, ^49^, ^50^, ^51^, ^52^, ^53^, ^54^, ^55^, ^56^, ^57^, ^58^, ^59^, ^60^, ^61^, ^62^, ^63^, ^64^, ^65^, ^66^, ^67^, ^68^, ^69^, ^70^, ^71^, ^72^, ^73^, ^74^, ^75^, ^76^, ^77^, ^78^ ^]^

## Conflict of Interests

The authors declare no conflict of interest.

## Supporting information



Supporting Information

## Data Availability

The data that support the findings of this study are available from the corresponding authors upon reasonable request.
