# Supplementary material for: Angularly Resolved Tip‐Enhanced Raman Spectroscopy
Source: Angew Chem Int Ed Engl. 2025 Jul 20;64(34):e202506152. doi: 10.1002/anie.202506152 (PMC12363624; doi:10.1002/anie.202506152)
Supplement: Supplementary file 1 — Supporting Information [file ANIE-64-e202506152-s001.pdf]

Supporting Information

Angularly Resolved Tip-Enhanced Raman Spectroscopy

Felix Schneider, [a+] Tim Parker, [a+] Liangxuan Wang, [a] Michel Rebmann, [a] Yang Zhao, [a] Eric Juriatti, [a] Heiko Peisert, [a] Alfred J. Meixner, [a] Johannes Gierschner, [c,a*] Lingyan Meng, [b*] Dai Zhang [a*]

[a] F. Schneider [+], T. Parker [+], L. Wang, M. Rebmann, Y. Zhao, E. Juriatti, H. Peisert, A. J. Meixner, D. Zhang
Institute of Physical and Theoretical Chemistry, Eberhard Karls University of Tübingen
Auf der Morgenstelle 15, 72076 Tübingen, Germany
E-mail: dai.zhang@uni-tuebingen.de

[b] L.-Y. Meng
School of physics and Physical Engineering, Qufu Normal University, P. R. China
E-mail: lymeng@qfnu.edu.cn

[c] J. Gierschner
Madrid Institute for Advanced Studies, IMDEA Nanoscience, C/Faraday 9, Ciudad Universitaria de Cantoblanco, 28049 Madrid, Spain
E-mail: johannes.gierschner@imdea.org

[+] These authors contributed equally to this work

1. TERS setup and excitation polarization

Stokes-Raman and anti-Stokes-Raman scattering experiments were performed on a home-built optical microscope. For the excitation, a 633 nm CW laser was focused onto the sample using a parabolic mirror with NA = 0.9986 (in air)[24] which also collected the optical signal. An Acton SpectraPro 300i spectrometer coupled to a Roper Scientific LN/CCD-1340/100-EB camera was used to collect Raman scattering and photoluminescence spectra. All Raman scattering spectra were collected using a 600 lines / mm grating with three spectra repetitions that were averaged. The acquisition time was 90 s for the energy-momentum spectroscopy. For a normal TERS spectrum the integration time was decreased to 30 s. For the anti-Stokes experiments, the Stokes and anti-Stokes scattering were simultaneously recorded with an acquisition time of 180 s. The laser intensity directly in front of the parabolic mirror was determined as 58 µW. A home-built mode-converter consisting of four quarters of a lambda-half waveplate was used to transform the linearly polarized laser beam into a radially or azimuthally polarized laser beam. After the mode conversion, a 20 µm pinhole was used as a mode cleaner.[25]

Either radially or azimuthally polarized laser beams were used to excite the tip with different electric field distributions in the focal field. A radially polarized laser beam focused with a NA = 0.9986 onto a gold film results in a focal field with an out-of-plane electric field intensity $|E_z|^2$ 24.6 times stronger than the in-plane-electric field intensity $|E_{x,y}|^2$. In contrast, in the center of the focal field of an azimuthally polarized laser beam under equal conditions possesses an exclusively in-plane electric field $|E_{x,y}|^2$. Furthermore, the electric field intensity of a radially polarized laser beam is the strongest in the center of the focal field, which is where the tip was positioned for the tip-enhanced optical measurements. For an azimuthally polarized laser beam however, the electric field intensity in the center of the focal field is zero, as it is located symmetrically on a ring about the optical axis. Therefore, the tip was moved by approximately 250 nm in x- or y-direction into the electric field intensity maximum of the focal field of the azimuthally polarized laser beam.

The gold tips used for this work were electrochemically etched from gold wires[26] with diameter a d = 127 µm and fixed onto a quartz tuning fork with a nominal resonance frequency of 32 kHz. The quartz tuning fork was mounted onto a piezo-tube and inserted into the parabolic mirror through a hole from above the sample. The tip was coarse positioned into the focus of the parabolic mirror using shear piezo stacks until a symmetric scattering pattern could be observed by an assisting camera. The sample was approached towards the tip by shear piezo chips. The nanometer-sized tip-sample distance was controlled and monitored during the experiment by the scanning probe microscopy controller (RHK Technology, SPM100), which used the demodulated phase shift signal generated by a lock-in amplifier (Ametek 7270 DSP) from the pre-amplified tuning fork signal.[27] The phase-shift could be adjusted gradually to achieve different tip-sample distances. Once the tip-sample shear force feedback was obtained, the tip-in-focus position was precisely optimized in the x-, y-, and z-directions until the maximum Raman scattering intensity is achieved.

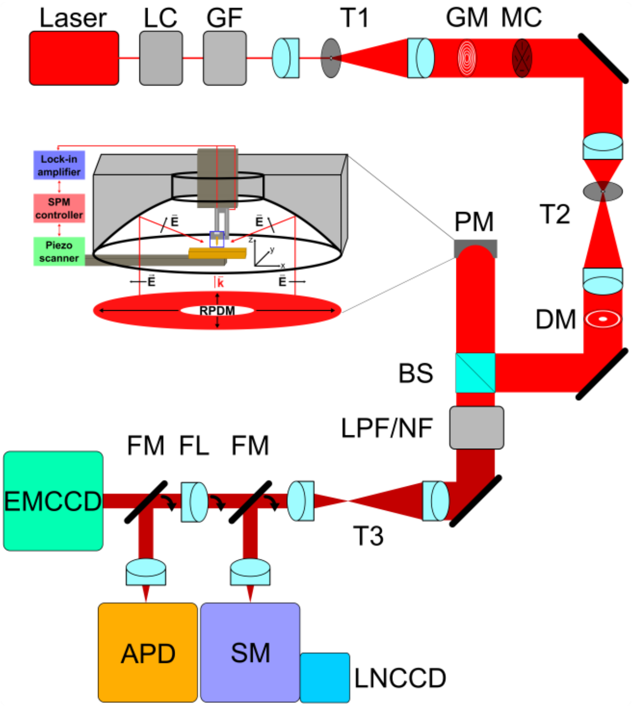

Figure S1: Home-built optical microscope setup for TERS measurements. A laser cleaning filter (LC) ensures the narrow emission line width at 633 nm. The laser intensity is adjusted with grey filters (GF). After telescope 1 (T1) the laser beam is expanded to fill the full aperture of the parabolic mirror (PM). The mode-converter (MC) transforms the Gaussian mode (GM) into a donut mode (DM), which is either radially or azimuthally polarized. The DM laser beam is further collimated by telescope 2 (T2) and purified with an additional pinhole as a mode cleaner. The beam is guided to the parabolic mirror (PM) using a 50/50 beam-splitter (BS). The sample is positioned in the focal point of the parabolic mirror, while the TERS tip is approached from the top (see magnified inset). The optical signal is collected by the PM and decreased in size in the third telescope (T3). A movable 2f-lens (FL) is positioned between the EMCCD and T3 so that the optical signals can be detected either for the back focal plane experiment or for other optical measurements. Two flipping mirrors (FM) are used to direct the beam path either to the imaging detector (APD) for optical imaging or the spectrometer (SM) coupled to a liquid-nitrogen cooled charged-coupled device (LNCCD) for spectroscopy.

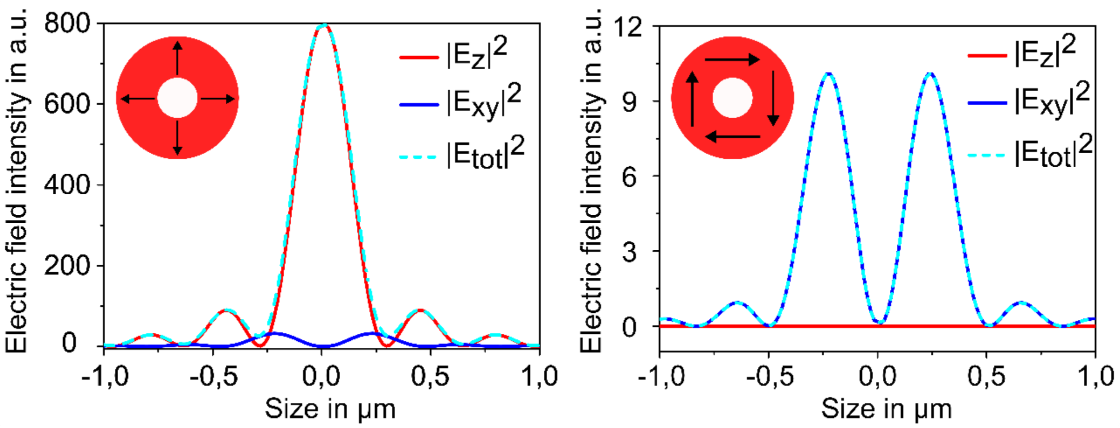

Figure S2: Electric field intensity in the focus of a radially polarized donut mode (RPDM, left) and an azimuthally polarized donut mode (APDM, right). The RPDM has a dominant out-of-plane electric field component ($|E_z|^2$) in the center of the focal field, and a minor in-plane electric field component ($|E_{xy}|^2$) in an annular shape about the center of the focal field. The APDM has no out-of-plane electric field component, only an in-plane electric field component in an annular shape about the center of the focal field.

2. Materials and sample preparation

Ultrasmooth gold films were produced by electron-beam physical vapor deposition on a polished Si(110) wafer following the descriptions in previous work.[28] The evaporation rate was 0.15 nm / s at a chamber pressure of $2 \cdot 10^{-6}$ mbar until a 100 nm thick gold layer was achieved. To obtain the ultrasmooth gold surface, a glass substrate was glued onto the gold layer with a UV-activated glue (Norland Optical Adhesive 63), which was pried off from the silicon wafer after 30 minutes of UV-irradiation and subsequent curing for 24 hours. Statistical sampling of the ultrasmooth gold surface using an atomic force microscope revealed an average root mean square of the surface roughness of 0.024 ± 0.001 nm (AFM contact mode, Veeco Nanoscope).

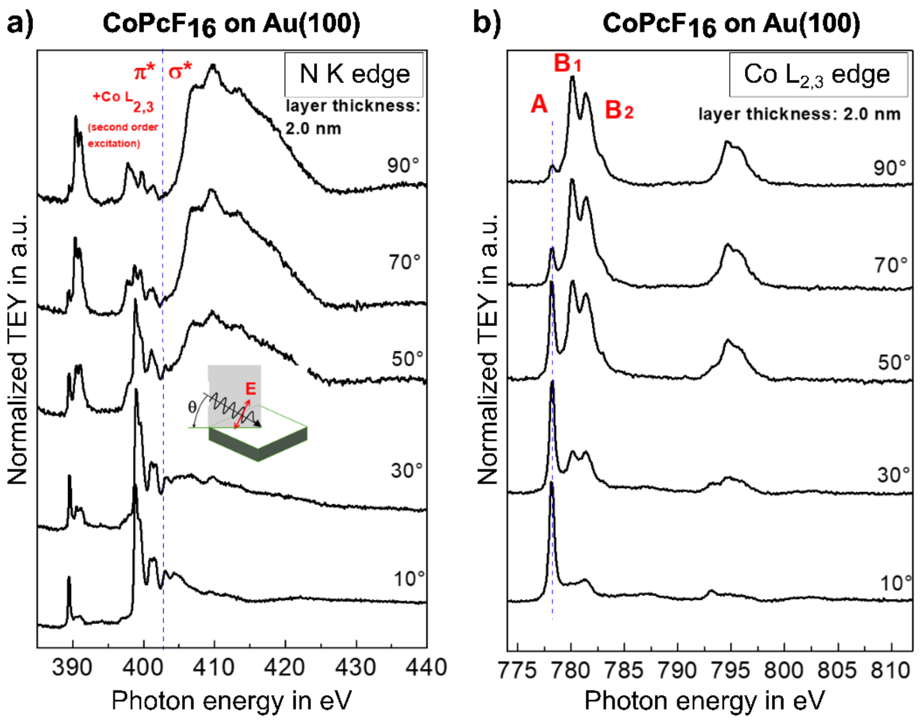

Figure S3: Polarization dependent X-ray absorption spectra (XAS) of a 2 nm thick $CoPcF_{16}$ film on Au(100) showing the flat-lying adsorption geometry. The spectra were taken in total electron yield (TEY) mode. Using linearly, p-polarized synchrotron radiation, at normal incidence (here, θ = 90°) we preferentially probe transitions into orbitals lying in the molecular plane (σ* in N K edge absorption spectra or B1 and B2 in Co L edge spectra), whereas at grazing incidence (θ = 10°) transitions into orbitals with out-of-plane components (π* and A) are maximal. This is clearly visible in a) and b), although p* features in a) are in superposition to second order excitations of the Co L edge.

The probe molecule $CoPcF_{16}$ was deposited by physical vapor deposition at ultra-high vacuum condition in a Knudsen cell at 450 °C. The films for Raman experiments were characterized by X-ray photoelectron spectroscopy (XPS) using a multi-chamber ultrahigh vacuum system equipped with a Phoibos 150 Hemispherical Energy Analyzer (SPECS), and an X-ray source with monochromator (XR 50 M SPECS). The template stripping procedure used to fabricate the ultrasmooth gold films leaves the substrate nonconductive, therefore the XPS measurements could not be performed directly on the gold films. An additional silicon wafer was mounted on the same sample holder close to the ultrasmooth gold films during the deposition of $CoPcF_{16}$, which was used for the characterization of the film. The layer thickness was estimated by comparing the molecule related intensities of the F 1s and N 1s core levels with substrate related Si 2s and Si 2p intensities (see Figure S3). Attenuation of the Si intensities by carbon contamination were taken into consideration via $I = I_0 \cdot e^{-\frac{d}{\lambda}}$ derived from Beer's law. The $CoPcF_{16}$ layer thickness was calculated accounting for the mean free path of photoelectrons following Seah and Dench,[29] and with photoionization cross-sections taken from Yeh and Lindau.[30] The Au(100) single crystal for XAS was prepared by repeated cycles of Ar ion sputtering and annealing. For details and a comparison to the angular dependence of Co L edge XAS spectra of CoPc, we refer to previous work.[31]

3. Distance-dependent geometries and Raman vibrational mode calculation

We performed electronic structure calculations at the Kohn-Sham DFT level using the CP2K/QUICKSTEP simulation package,[32] in which Gaussian and plane wave (GPW) formalism is implemented. The auxiliary PW basis used to represent the valence electron density in reciprocal space has an energy cutoff of 600 Ry. In all cases, the exchange-correlation PBE[33] functional was employed using the DZVP-MOLOP-SR-GTH Gaussian basis set with a D3 dispersion correction.[34] This level of theory is sufficiently accurate to reproduce the reconstruction of the Au(111) surface, in good agreement with previous works.[35] The Au surface was described via a slab model, with a lattice parameter of 4.08 Å.[36] To prevent any interaction between periodically replicated slabs, 20 Å of vacuum space were added along the surface normal. The $CoPcF_{16}$/Au system consisted of a gold cluster Au_{10} as a tip, a probe $CoPcF_{16}$ and a 9 × 9 atom Au(111) slab, including four layers, of which the bottom two were fixed. Dipole correction was applied along the z-direction. This correction is essential for reliably describing the electrostatic potential and electronic states of asymmetric slab systems with molecular adsorbates, as previously shown for open-shell systems on metal substrates. The overall system was constructed as a singlet, while $CoPcF_{16}$ retained its doublet configuration (S = 1/2) in the presence of a sufficient number of gold atoms, ensuring natural spin and charge compensation in the model. Only the gamma point was sampled in reciprocal space. In order to reveal the tip distance dependent geometries, the tip-substrate distance was systematically reduced from 10 Å to 6 Å. The strong Coulomb interactions between the Co and Au atoms break the planarity of the $CoPcF_{16}$ backbone, reducing the symmetry from D4h to C4v.

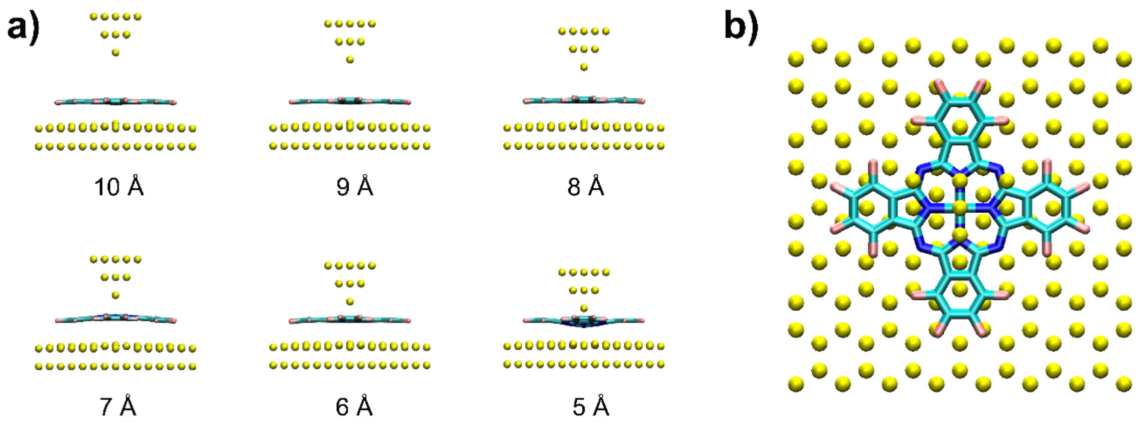

Figure S4: Calculated geometries of tip-$CoPcF_{16}$-substrate configuration with tip distance decreasing from 10 Å to 6 Å. **a)** For small tip-sample distances, the strong Coulomb interactions between the Co and Au atoms break the planarity of the $CoPcF_{16}$ backbone, reducing the symmetry from D_{4h} to C_{4v}. For simplification, only the top two layers of gold atoms are shown. **b)** Top-view of the tip-$CoPcF_{16}$-substrate configuration.

With the optimized geometries, frequency calculations of the probe $CoPcF_{16}$ were further performed in the Gaussian16 program package[37] to obtain a better description. All Au atoms were replaced by restrained electrostatic potential (RESP) background charges derived from CP2K calculations. This strategy mimics the dielectric response of the metallic substrate and tip during the Gaussian-based vibrational analysis, allowing $CoPcF_{16}$ to experience a realistic electrostatic

environment without the computational cost of treating explicit metal atoms in the quantum region. Specifically, RESP charges from CP2K have shown superior electrostatic potential reproducibility.[38] Herein, the dispersion-corrected D3-B3LYP functional with the standard 6-31G(d) basis set for C, N, F and the LanL2TZ basis set for Co was applied. Besides, a free $CoPcF_{16}$ in vacuum was computed for comparison. Raman spectra were computed on the free structure with a frequency scaling factor of 0.995. A detailed protocol was reported in our recent work.[39]

To better reproduce the resonance Raman scattering, an empirical energy offset of 0.15 eV was applied. This value was determined by comparing the TD-DFT computed energy of the first bright excited state of $CoPcF_{16}$ in vacuum (2.01 eV, 617 nm) to the experimental absorption maximum recorded in THF (1.86 eV, 665 nm).[40] Hence, the excitation wavelength of 633 nm (1.95 eV) in the experiment corresponds to an excitation wavelength of 590 nm (2.10 eV) in the calculations, which places the calculation safely in the resonance regime and ensures the validity of the polarizability derivative method used for the Raman intensity simulation. As shown in Figure S5, calculated Raman spectra at the same excitation wavelength (590 nm in simulation, corresponding to 633 nm in experiment) exhibit significant differences depending on the model: (a) free molecule; (b) distorted $CoPcF_{16}$ at a tip distance of 10 Å, modeled with RESP background charges; (c) distorted $CoPcF_{16}$ at a tip distance of 6 Å using the pre-resonance Raman treatment; and (d) distorted $CoPcF_{16}$ at 6 Å using the resonance Raman treatment, which additionally involves the excited-state energy gradient as required for resonance Raman simulations. Remarkable differences are shown between (c, d) $CoPcF_{16}$ at the distance of 6 Å and (a) free molecule, while there is almost no change observed for (b) a distance of 10 Å. This is due to the fact that, on one hand, the background charge provides a more realistic dielectric environment, which is reflected on the reproducible resonance Raman at 590 nm, corresponding to 633 nm in experiments; on the other hand, the distortion in geometries has a pronounced influence on the molecular electronic descriptions, hence affects the induced dipole moment within the molecule based on its polarizability. Specifically, this reduction in molecular symmetry activates certain Raman modes in the distorted geometry, namely, the out-of-plane vibrational mode at 678 cm^{-1} (calculated at 722 cm^{-1}) and at 740 cm^{-1} (collective modes, calculated around 780 cm^{-1}). Apart from that, as shown in Table S1, the intensity of some in-plane modes, for instance, 1372 cm^{-1} (calculated at 1355 cm^{-1}) drastically changes.

To illustrate the necessity of resonance Raman treatment and to clarify the differences between pre-resonance and resonance regimes, we compare both regimes at different excitation wavelengths in Figure S5 and Table S1. The pre-resonance Raman spectra shown in panels (a–c) of Figure S5 were obtained based on the ground-state geometry and the corresponding frequency analysis at different excitation wavelengths (non-resonance, 530 nm, 590 nm, and 633 nm). This approach assumes that the molecular geometry remains unchanged under photoexcitation and does not involve excited-state gradients. In contrast, the resonance Raman spectrum in panel (d) was computed using both the optimized ground-state geometry and the corresponding excited state. In all cases, the equilibrium geometries were confirmed to be stationary points on the respective potential energy surfaces by the absence of imaginary frequencies, validating the harmonic approximation for the vibrational analysis. As shown in panel (d), the resonance Raman approach yields improved agreement with the experimental spectra, particularly in reproducing key spectral features and intensity trends. This supports the reliability of vibrational mode assignments across excitation wavelengths and validates the necessity of considering excited states under on-resonance conditions. Overall, this combination of CP2K and this combination of CP2K and Gaussian calculations allowed for a fast and efficient optimization procedure combined with accurate frequency calculations.

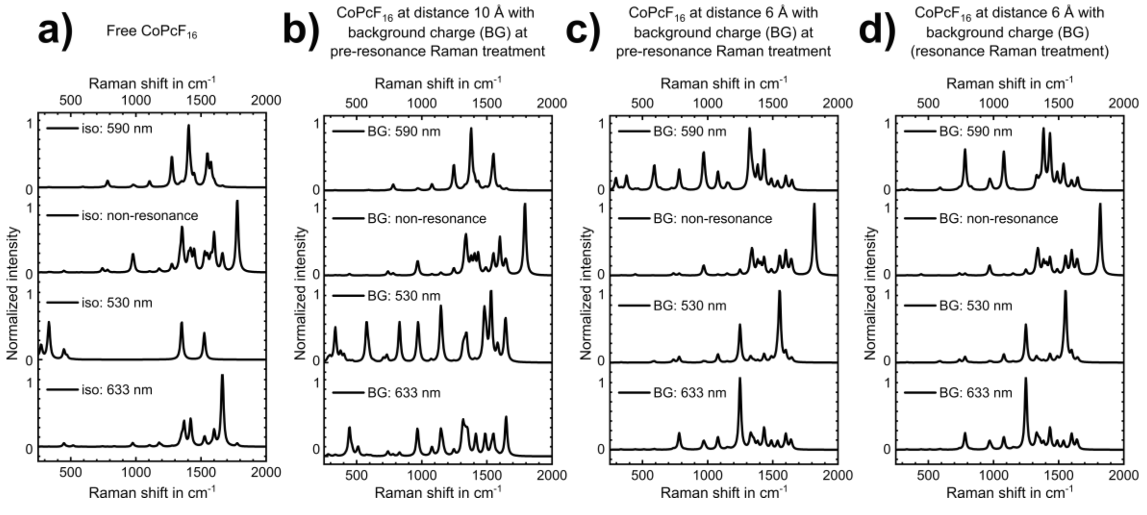

Figure S5: Calculated Raman spectra for **a)** free $CoPcF_{16}$, **b)** distorted $CoPcF_{16}$ at distance 10 Å (within the pre-resonance Raman treatment), **c)** distorted $CoPcF_{16}$ at distance 6 Å (within pre-resonance Raman treatment) and **d)** distorted $CoPcF_{16}$ at distance 6 Å (within the resonance Raman treatment) at different excitation wavelengths (590 nm in the calculation corresponds to 633 nm in the experiment).

Table S1: Calculated Raman activities of frequencies of interest (out-of-plane mode at lower frequencies 720-780 cm^{-1}, and in-plane mode at higher frequencies 1354 – 1370 cm^{-1}) for free $CoPcF_{16}$ and distorted $CoPcF_{16}$ at distances 10 Å and 6 Å within pre-resonance and resonance treatments following the Raman spectra in Figure S5. The excitation wavelength of 633 nm in the experiment corresponds to an incident wavelength of 590 nm in the calculations.

Compound	Mode	Frequency (cm^{-1})	Raman Intensity (Å^4/amu)			
			Non-resonance	530 nm	590 nm	633 nm
Free $CoPcF_{16}$	Mode 92	720	0	0	0	0
	Mode 97	776	0	0	0	0
	Mode 136	1370	152	0	24750	4692301
$CoPcF_{16}$ at distance 10 Å (pre-resonance)	Mode 92	721	1	49957	272	72367
	Mode 99	779	1	0	56043	287734
	Mode 136	1354	181	0	40864	4587133
$CoPcF_{16}$ at distance 6 Å (pre-resonance)	Mode 92	722	2	3158	252008	5377
	Mode 99	779	2	9598	108495	4491
	Mode 136	1355	35	175909	826137	712006
$CoPcF_{16}$ at distance 6 Å (resonance)	Mode 92	722	2	5377	16164	3158
	Mode 99	779	2	4491	42145	9598
	Mode 136	1355	35	175909	588658	590128

4. Gap-plasmon resonance

The plasmonic resonance of the tip-sample configuration highly depends on the individual gap geometry. It is a well-known challenge for TERS experiments that each tip will affect the obtained experimental results, either through the coupling strength or the wavelength of the plasmonic resonance of the gap-mode. In TERS spectra, the plasmonic gap-resonance can be extracted from the gold photoluminescence background.[41] While the tip used for the experiments shown in Figure 2 only allows for the detection of anti-Stokes Raman scattering up until the peak at -967 cm^{-1}, other tips with a broader resonance or a resonance maximum shifted to shorter wavelengths make it possible to obtain anti-Stokes Raman scattering up until the peak -1540 cm^{-1}. An example is shown in Figure S6a, although the intensity and resolution of these peaks is low. For the distinct tip geometry used in the experiments shown in the main text, the gap-plasmon resonance is calculated at λ = 640 nm, see Figure 3b. This is in good agreement with the experimentally obtained maximum of the gold photoluminescence background at λ = 651 nm to λ = 656 nm (see Figure S6b), depending on the tip-sample distance.

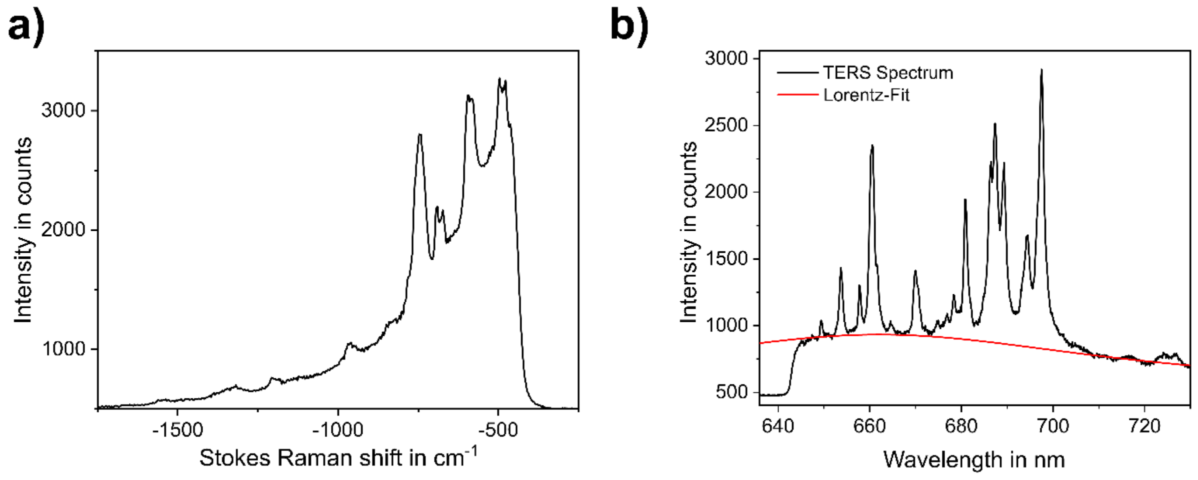

Figure S6: Influence of the plasmon resonance of the gap-plasmon. **a)** Anti-Stokes Raman peaks up to -1540 cm^{-1} can be seen in TERS experiments using different tips than the one shown in the main text. **b)** Fitting of the photoluminescence background using a Lorentzian function. The Raman peaks and the filter cutoff region are masked during the fitting procedure.

5. Simulated directed emission

To better understand the experimental phenomenon, the finite-difference time-domain (FDTD) method was used to calculate the far-field scattering spatial distribution of TERS. Using the near-field to far-field transformation method[42], the angle-resolved emission pattern of TERS can be obtained based on the simulated near-field distribution at the transformation surface, which is parallel to the Au film and above the tip. The TERS model consists of a slightly tilted gold tip above an Au film. The tilting angle with respect to the optical axis (i.e., the sample normal), cone opening angle and apex diameter of the Au tip are α = 3°, β = 18° and R = 70 nm, respectively, which were determined from scanning electron microscope images from the used tip. $CoPcF_{16}$ thin film with a thickness of 1 nm locates on the surface of the Au film. The Au tip-$CoPcF_{16}$ molecule distance is changed from 1 nm to 4 nm. An electric dipole source was used to model the excited molecule and was placed in the center of the molecular layer. A truncated tip with the length of 500 nm was used in all calculations to save the computation resource without sacrificing the accuracy. A perfectly matched layer condition was used for all boundaries of the rectangular simulation region with a size of 10 μm × 10 μm × 10 μm. A non-uniform mesh with a mesh size of 0.2 nm was used to accurately simulate the narrow tip-film nanogap while the mesh size at the tip apex was 0.5 nm. The optical constant for Au and $CoPcF_{16}$ molecule were taken from experimental reports.[43]

The angle-resolved far-field emissions at different d were calculated to understand the far-field signal formation from the tip-sample gap. The scattered electric field in the far-field can be obtained through the near-field to far-field method which is expressed by[42]

$$\begin{aligned} E_\theta &= \frac{exp(-jkr)}{4\pi r}(-jk)(Zf_\theta + f_{m\,\varphi}) \\ &= -jk\frac{exp(-jkr)}{4\pi r}\left[Z\left(f_x\,cos\theta\,cos\varphi + f_y\,cos\theta\,sin\varphi - f_z\,sin\theta\right) + \left(-f_{m\,x}\,sin\varphi + f_{m\,y}\,cos\varphi\right)\right] \end{aligned} \tag{1}$$

$$\begin{aligned} E_\varphi &= \frac{exp(-jkr)}{4\pi r}(jk)(-Zf_\varphi + f_{m\,\theta}) \\ &= jk\frac{exp(-jkr)}{4\pi r}\left[Z\left(f_x\,sin\varphi - f_y\,cos\varphi\right) + \left(f_{m\,x}\,cos\theta\,cos\varphi + f_{m\,y}\,cos\theta\,sin\varphi - f_{m\,z}\,sin\theta\right)\right] \end{aligned} \tag{2}$$

Here, $Z = \sqrt{\mu/\varepsilon}$ is the wave impedance in free space. The r is the position of the field point and k is the propagation constant in free space. In cartesian coordinates, we have the electric and magnetic current moments f_λ and $f_{m\,\lambda}$:

$$f_\lambda = \int_{S'} J_\lambda(r')\,exp\left(jkx'\,sin\theta\,cos\varphi + jky'\,sin\theta\,sin\varphi + jkz'\,cos\theta\right)ds' \tag{3}$$

$$f_{m\,\lambda} = \int_{S'} J_{m\,\lambda}(r')\,exp\left(jkx'\,sin\theta\,cos\varphi + jky'\,sin\theta\,sin\varphi + jkz'\,cos\theta\right)ds' \tag{4}$$

Here, $\lambda = x, y, z$ represent the unit vectors of the three components of the cartesian coordinates, and r' is the position of the source point. The equivalent electric and magnetic currents $\vec{J}_\lambda$ and $\vec{J}_{m\,\lambda}$ can be obtained from the output plane (S') with electromagnetic field distribution in near field, and are expressed by

$$\vec{J}_\lambda = \vec{e}_n \times \vec{H} \tag{5}$$

$$\vec{J}_{m\,\lambda} = -\vec{e}_n \times \vec{E} \tag{6}$$

where $\vec{E}$ and $\vec{H}$ are the surface electric and magnetic fields. The $\vec{e}_n$ is the normal unit vector of the output plane.

Eq. (1) and Eq. (2) indicate that the calculated far-field emissions are related to the polar angle (θ) and azimuthal angle (φ), which are shown in the main text Figure 3a. The calculated results are shown in Figure 3c–f in the main text and in Figures S7 and S8. Understanding the tip-molecule interaction is of importance to the optimal design of TERS configuration with high detection sensitivity and collection efficiency of Raman signal. A two coupled driven oscillators model can be used to interpret the tip plasmon-molecular exciton interaction, which is described by.[44]

$$\begin{pmatrix} \omega_p^2 - \omega^2 + i\omega\gamma_p & -g_e^2 \\ -g_p^2 & \omega_e^2 - \omega^2 + i\omega\gamma_e \end{pmatrix}\begin{pmatrix} A_p \\ A_e \end{pmatrix} = \begin{pmatrix} f_p \\ f_e \end{pmatrix} \tag{7}$$

Here, the ω_m, γ_m and f_m are the resonant frequencies, damping coefficients and the amplitudes of the external forces, respectively, with the driving frequency ω. Subscript *m* = *p* represents the tip plasmon and *m* = *e* represents the molecule emitter. $A_p$and A_e are the oscillator amplitudes and can be written as:

$$A_p = \frac{\left(\omega_e^2 - \omega^2 + i\gamma_e\omega\right) f_p + g_e^2 f_e}{\left(\omega_p^2 - \omega^2 + i\gamma_p\omega\right)\left(\omega_e^2 - \omega^2 + i\gamma_e\omega\right) - g_e^2 g_p^2} \quad (8)$$

$$A_e = \frac{\left(\omega_p^2 - \omega^2 + i\gamma_p\omega\right) f_e + g_p^2 f_p}{\left(\omega_p^2 - \omega^2 + i\gamma_p\omega\right)\left(\omega_e^2 - \omega^2 + i\gamma_e\omega\right) - g_e^2 g_p^2} \quad (9)$$

The relation between the f_e and f_p is $f_e = Mf_p$, where *M* is the plasmon-enhanced factor. The tip–molecule interaction can be described by the coupling strengths g_p and g_e by $g_p^2 = Mg_e^2$. Strong coupling between the tip plasmon and molecular exciton requires that the damping coefficients of both oscillators are weak, $|\gamma_e| \ll |g_e|$ and $|\gamma_p| \ll |g_p|$, which shows Rabi splitting in the spectra. In the weak-coupling regime, the damping coefficient is much stronger than one of the coupling strengths, $|\gamma_p| \gg |g_e|$ or $|\gamma_e| \gg |g_p|$. Here, our experimental system is in the weak-coupling regime where no Rabi splitting behavior can be observed in our calculations.

The far-field emission of the optical signal emerging from the plasmonic gap depends on the wavelength of the emitted light, as indicated in Eq. (8) and Eq. (9). Two significant changes need to be considered depending on the emitted wavelength: 1) The coupling to the gap-plasmon decreases as the emitted wavelength is shifted away from the plasmonic gap resonance maximum, shown in Figures S7a and S8a. This leads to a decreased intensity of the radiation due to weaker enhancement. 2) The angular emission pattern is strongly influenced by the wavelength of the emitted light. As the wavelength increases from λ = 640 nm to λ = 720 nm, the maximum emission angle changes from θ = 40 ° to θ = 31 ° for a vertical dipole (Figure S7b), and from θ = 42 ° to θ = 35 ° for a parallel dipole (Figure S8b). Additionally, the symmetry of the vertical dipole far-field emission changes from a rather homogeneous φ angle emission at λ = 640 nm to an emission towards a distinct φ angle-range (here: towards φ = 90 °, see Figure S7b). The φ-directionality persists for all wavelengths for a parallel dipole, although the relative intensity distribution is spread to a slightly larger φ angle-range when the wavelength increases, see Figure S8b.

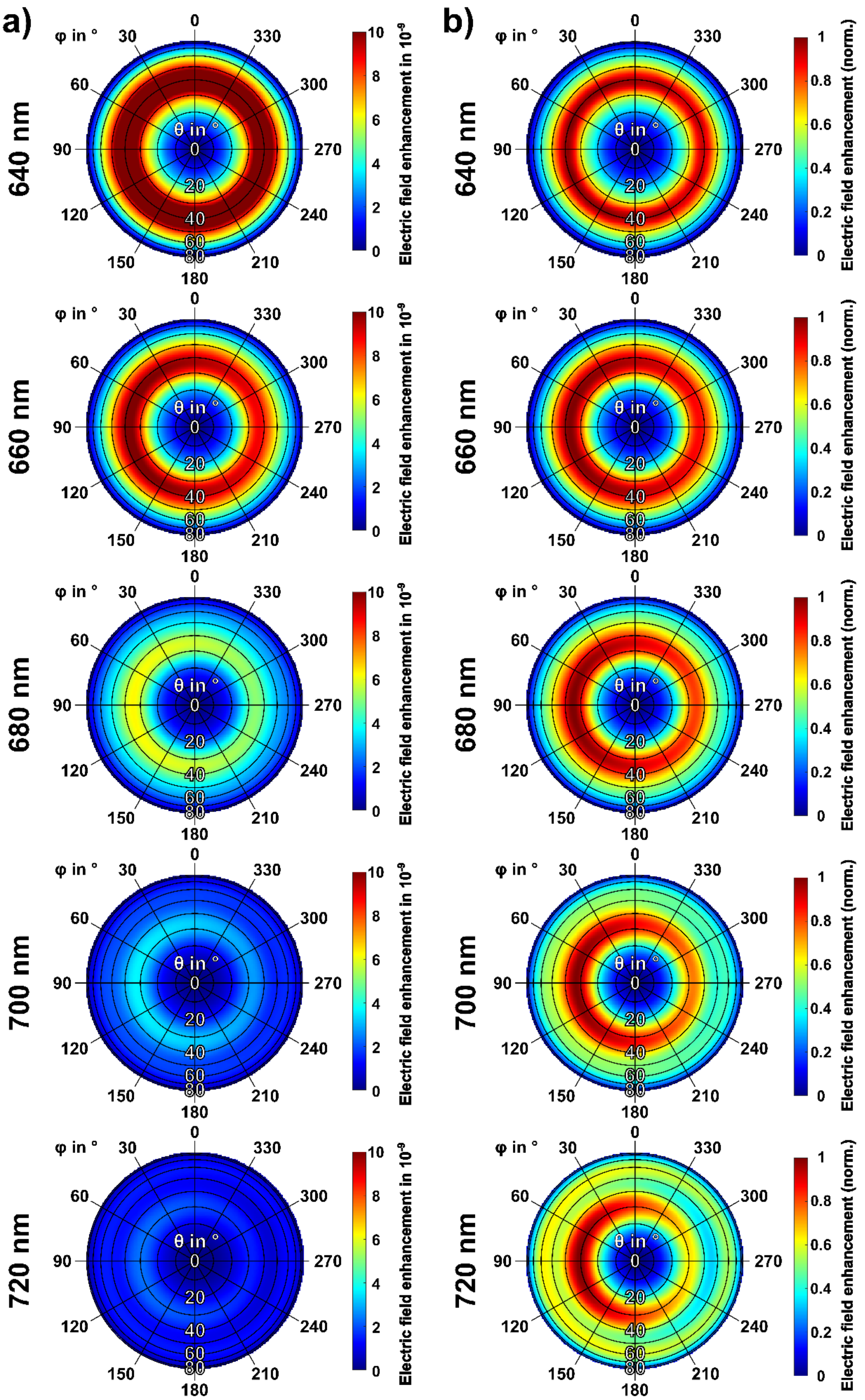

Figure S7: Influence of emission-wavelength on the far-field emission pattern of a perpendicular dipole in the gap. **a)** Emission pattern for λ = 640-720 nm in steps of 20 nm from top to bottom. **b)** The same as a), but with normalized intensity for each image scale to highlight the changes in directionality.

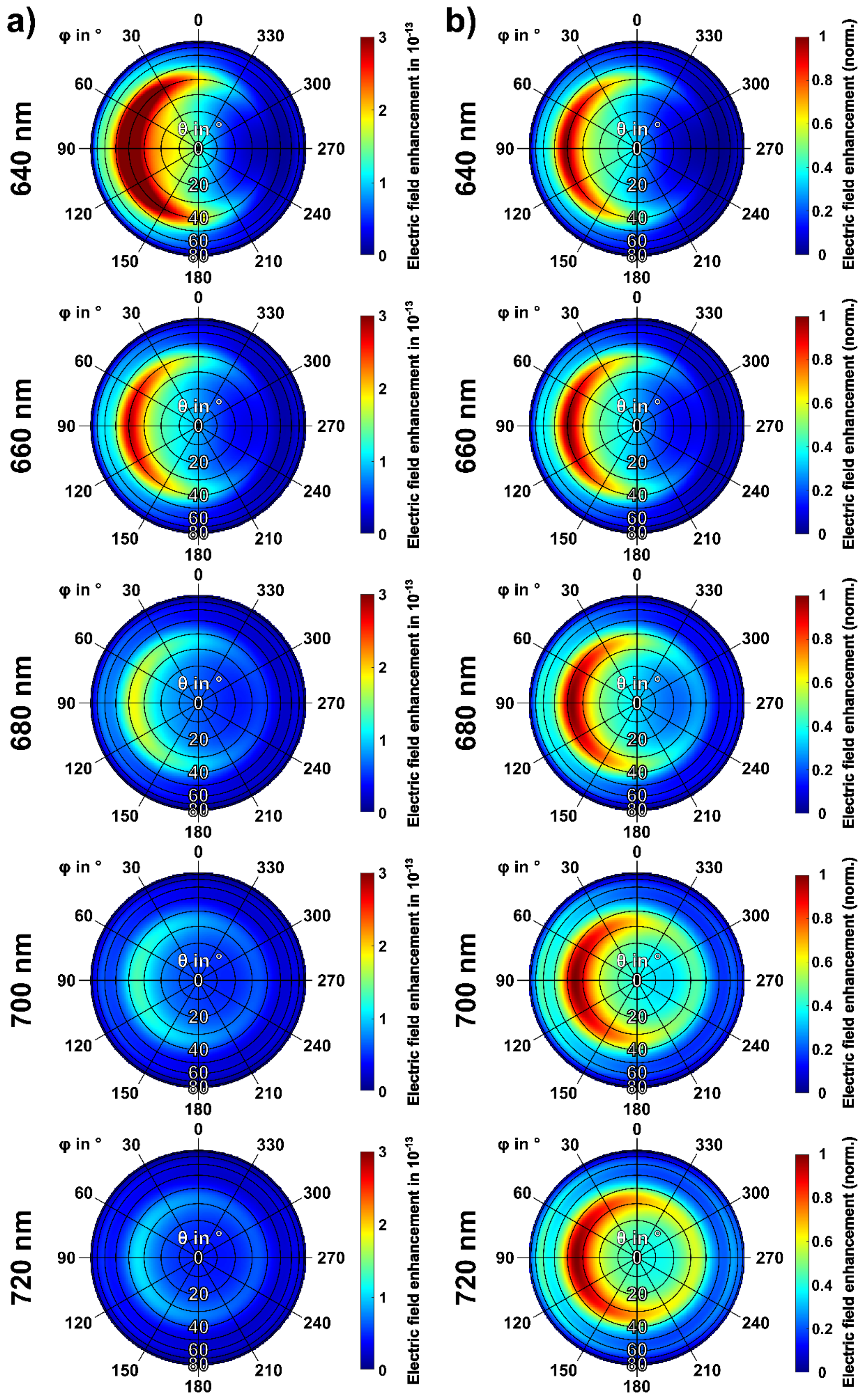

Figure S8: Influence of emission-wavelength on the far-field emission pattern of a parallel dipole in the gap. **a)** Emission pattern for λ = 640-720 nm in steps of 20 nm from top to bottom. **b)** The same as a), but with normalized intensity for each image scale to highlight the changes in directionality.

The far-field emission pattern strongly depends on the tilting angle of the tip. Figure S9 shows a comparison of the simulated electric field enhancement in the far-field of the experimental tilting angle α = 3° compared to a tilting angle of α = 0°. While the radiation pattern at α = 0° is rather symmetric about the optical axis, that at α = 3° is highly directional towards φ = 90° away from the tip tilting direction (tilted towards φ = 270°). However, the position of the tip above the simulated emitter dipole only very weakly influences the radiation pattern, see Figure S10. For a dipole vertical to the sample surface, the tip position only changes the relative intensity slightly. Similar results emerge for the dipole parallel to the sample surface, where the maximum emission angle remains unchanged, however a small side maximum emerges in the range of θ = 0° to -15°.

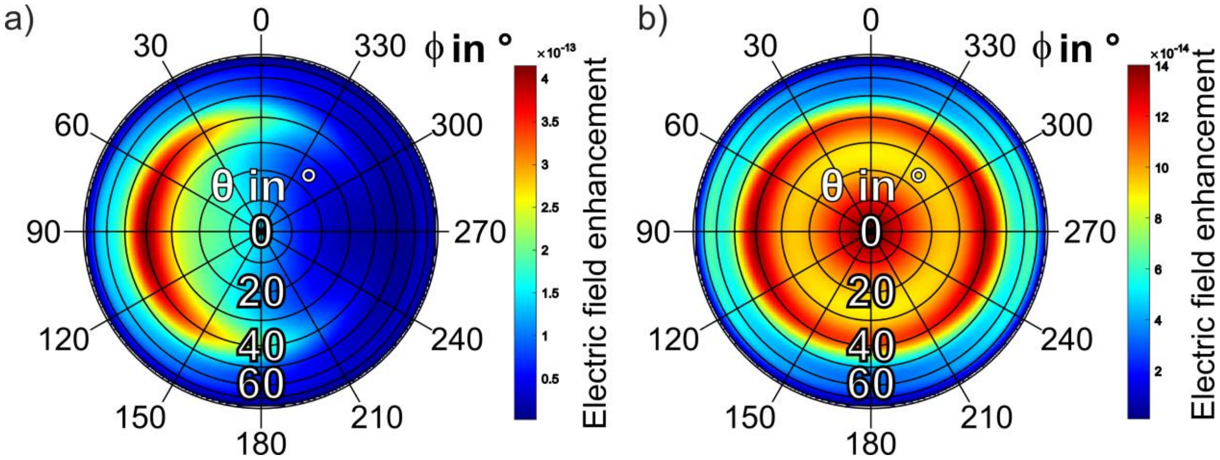

Figure S9: Radiation patterns for λ_{em}= 640 nm at d = 2 nm simulated for an emitter dipole parallel to the sample surface. Left: A tilted tip with a tilting angle α = 3 °; Right: An upright tip with α = 0 °. The tip apex opening angle β and the apex diameter R are simulated as α = 3 °, β = 18 ° and R = 70 nm, respectively.

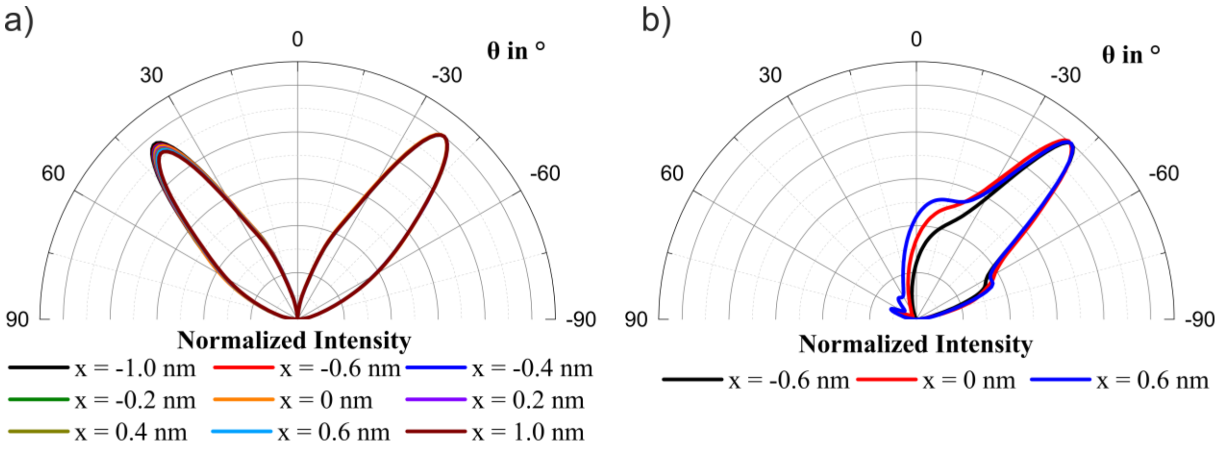

Figure S10: Normalized emission intensity radiation patterns for λ_{em}= 640 nm at d = 2 nm simulated for an emitter dipole at z = 0 **a)** vertical and **b)** parallel to the sample surface. The emitter is positioned off-axis in x-position (distances away from the field maxima) as indicated. The tip tilting angle vs. the optical axis α, the tip apex opening angle β and the apex diameter R are simulated as α = 3 °, β = 18 ° and R = 70 nm, respectively.

6. Collection efficiency

The parabolic mirror used in this work allows for the collection of all optical signal in the angle-range from θ = 27° to θ = 87° for all angles of φ, minus the portion of the aperture blocked by the sample holder bridge. Compared to commonly used side-illumination TERS configurations, the amount of optical signal collected by our parabolic mirror is significantly higher. More importantly, the parabolic mirror allows us to collect optical signals from all φ angles, which is a strong limitation for side-illumination configurations. The typical numerical aperture for side-illumination TERS experiments is small at NA ≈ 0.5 for the sake of obtaining a long working distance.[45] Regarding the discussion of angularly resolved TERS experiments, it is critical to illustrate the impact of the numerical aperture for signal collection. In addition to the calculations of tip-sample gap-plasmon emission patterns, we have overlayed calculations of the collection efficiency for

side-illumination TERS with the experimental configuration used for previous milestone experiments, where the objective with NA ≈ 0.5 is positioned at θ = 60° with respect to the optical axis.[45b] We note that the wavelength of the emission strongly influences the emission pattern, and therefore for detailed analysis of experimental results the respective wavelength-regions must be considered (see Supporting Information 5). Here we show the calculations for λ = 680 nm as an example, which represents the middle of our experimental wavelength range.

We consider both extreme cases for the emission of a dipole perpendicular to the sample surface and parallel to the sample surface coupled to the gap-plasmon. For the perpendicular case, the emission pattern is more symmetric about the φ angle than for the parallel case, resulting in lower collection efficiency discrepancies. When the objective is positioned at the φ angle of maximum emission intensity, 14.9 % of the total emission is collected, while on the opposite side 12.6 % of the total emission is collected (Figure S11a). In other words, there is a systematic influence on the obtained spectral intensity that can explain variations of TERS spectral intensity of up to 18.3 %, simply determined by the experimental setup at which φ angle the collection aperture is positioned. This effect is even stronger for the case of a parallel dipole emission coupled to the gap-plasmon, where the aperture position at an optimal angle leads to a collection efficiency of 20.5 %, while on the opposite side merely 6.4 % of the total emission is collected (Figure S11b). Thus, the observed spectral intensity of an in-plane dipole could vary up to 320.3 % depending on the φ angle at which the objective is positioned. We emphasize that side-illumination TERS configurations could therefore be prone to strong variations of relative Raman intensity among different spectra collected on the same sample, influenced by the molecular orientation, the tip-geometry and tip tilting angle, and by the objective position in the experiment.

To calculate the fraction of the emitted intensity that is collected by the objective at θ_{obj} = 60°, one must calculate the intersection circle of the cone spanned by the collection angle of the objective θ_{NA} and the unit-hemisphere which was used to visualize the simulated radiation. As a first step, the center point of this intersection circle must be calculated, which can be done through two known points on the intersection-face $\theta = \theta_{obj} \pm \theta_{NA}$:

$$\overrightarrow{r_{cen}} = \begin{pmatrix} \frac{\sin(\theta_{obj} - \theta_{NA}) + \sin(\theta_{\text{obj}} + \theta_{NA})}{2} \cdot \cos(\phi) \\ \frac{\sin(\theta_{obj} - \theta_{NA}) + \sin(\theta_{\text{obj}} + \theta_{NA})}{2} \cdot \sin(\phi) \\ \frac{\cos(\theta_{obj} - \theta_{NA}) + \cos(\theta_{obj} + \theta_{NA})}{2} \end{pmatrix}$$

The radius of the sphere defining the intersection is $\sin(\theta_{NA})$ leading to the following equation to describe all points within the intersection area:

$$|\vec{r} - \overrightarrow{r_{cen}}| \leq \sin(\theta_{NA})$$

For any point on the unit hemisphere $\vec{r}$ which fulfills this equation, the simulated intensity is collected by the objective. Any point outside of this area is not collected by the objective.

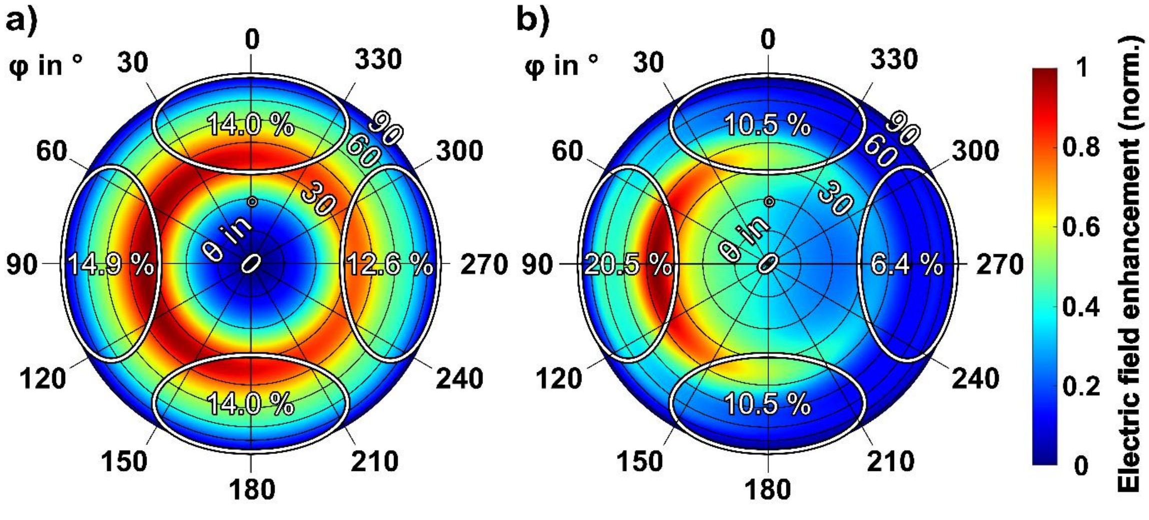

Figure S11: Collection efficiency with a NA = 0.5 air-objective positioned at θ = 60° with respect to the optical axis. **a)** Shows the collection efficiency for a perpendicular dipole coupled to the tip-sample gap-plasmon at four distinct φ angles. **b)** The same as a) but for a parallel dipole.

7. Angularly resolved TERS experiment

To measure the angularly distributed Raman scattering, we used the home-built parabolic mirror assisted setup shown as Figure S1. A collimated continuous-wave laser with a wavelength of 633 nm is focused by the parabolic mirror onto the sample, which also collects the optical signal from the exact focal point. The optical signal is reflected by the parabolic mirror, and parallelly transmits to a telescope. A 2f lens (f = 150 mm) is placed after the telescope, creating a first image plane of the parabolic mirror back-focal plane.[46] A slit with 200 µm width is placed inside the telescope to allow a subsection into five equally sized portions of the back-focal plane image.[46] To reconstruct the radiation pattern, the slit position is precisely moved using a kinematic stage from left to right in order to select vertical columns, and from top to bottom to select horizontal rows of the back-focal plane image. The optical signal for each column is then dispersed separately by the spectrometer's grating onto a CCD so that the angular scattering can be spectrally resolved.

Table S2: Experimental angular subdivision for the investigated back-focal plane sections. The upper part of the table displays the Slices 1 to 5 using a horizontally positioned slit, the lower part of the table displays the Slices 6 to 10 using a vertically positioned slit. Given are the boundary lines defined by two points on the back-focal plane image between the sections following the illustration in Figure 4a and Figure S12 in coordinates of φ only, since θ = 87° for all of them.

Spatial subdivision	1st boundary line coordinates (φ) in °	2nd boundary line coordinates (φ) in °
Slice 1	Line A from A_1(195°) to A_2(356°)	Line B from B_1(179°) to B_2(12°)
Slice 2	Line B from B_1(179°) to B_2(12°)	Line C from C_1(153°) to C_2(38°)
Slice 3	Line C from C_1(153°) to C_2(38°)	Outer edge of the aperture
Slice 4	Line D from D_1(219°) to D_2(332°)	Line A from A_1(195°) to A_2(356°)
Slice 5	Line D from D_1(219°) to D_2(332°)	Outer edge of the aperture
Slice 6	Line E from E_1(80°) to E_2(289°)	Line F from F_1(105°) to F_2(264°)
Slice 7	Line F from F_1(105°) to F_2(264°)	Line G from G_1(130°) to G_2(239°)
Slice 8	Line G from G_1(130°) to G_2(239°)	Outer edge of the aperture
Slice 9	Line H from H_1(54°) to H_2(315°)	Line E from E_1(80°) to E_2(289°)
Slice 10	Line H from H_1(54°) to H_2(315°)	Outer edge of the aperture

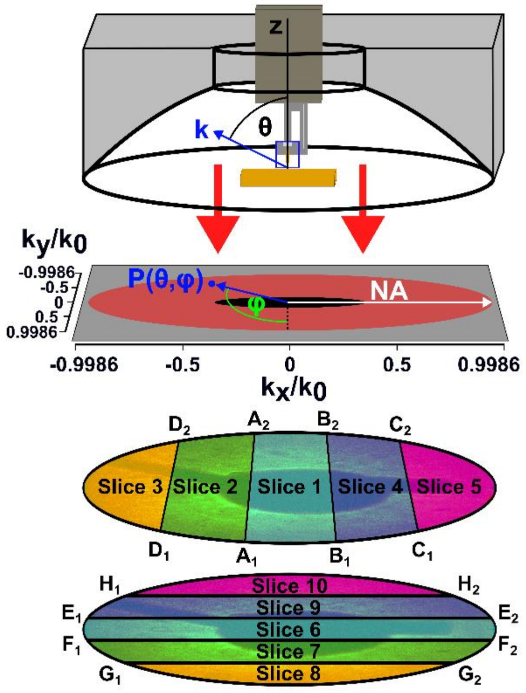

Figure S12: Experimental setup for the back-focal plane sectioning. The numerical aperture of the parabolic mirror limits the acquired k-space image. The center part marked in black cannot be detected due to the hole in the parabolic mirror. The division into multiple sections is illustrated as lines between a starting point and end point on the outer edge. Precise coordinates are provided in Table S2.

8. Normalized angularly resolved TERS emission intensity at different gap distances

To investigate the influence of the tip-sample distance on the emission directionality of the gap-plasmon, we performed the experiments for a small gap shown in Figure 4g and 4i also for a medium and large gap. The data analysis followed the same procedure as for the small gap described in the main text. Overall, the normalized intensity of the Raman peaks of the out-of-plane and in-plane vibration are only slightly changed as compared to the small gap. There are small changes observed in Slices 1, 4 and 8, where the difference between in-plane and out-of-plane vibrations increases in Slice 1 when the gap distance is decreased. For Slice 4 and 8, the difference between in-plane and out-of-plane vibrations decreases when the gap distance is increased. The small impact of the gap-distance on the angular distribution of Raman scattering is in line with the FDTD simulations shown in Figure 3c and 3d, where the simulated maximum emission angle changes only slightly for the dipole parallel to the sample surface, and remains unchanged for the dipole vertical to the sample surface.

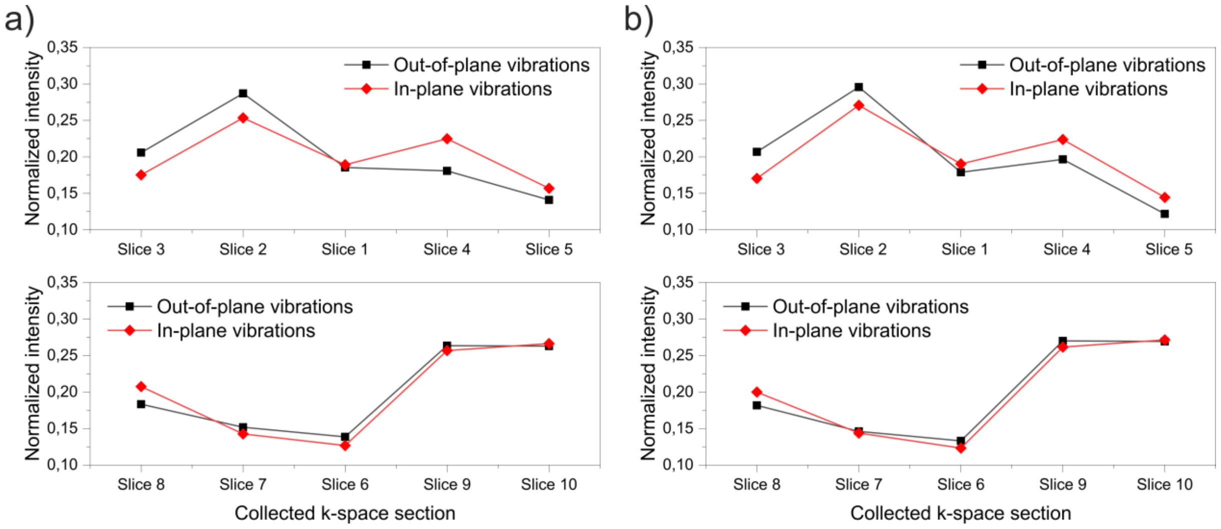

Figure S13: Normalized angularly resolved TERS emission intensity for in-plane and out-of-plane vibrational modes at **a)** large gap distance, and **b)** medium gap distance.

[24] a) A. Drechsler, M. Lieb, C. Debus, A. Meixner, G. Tarrach, *Opt Express* **2001**, *9*, 637-644; b) M. Lieb, A. Meixner, *Opt Express* **2001**, *8*, 458-474; c) J. Stadler, C. Stanciu, C. Stupperich, A. J. Meixner, *Opt Lett* **2008**, *33*, 681-683.

[25] R. Dorn, S. Quabis, G. Leuchs, *Phys Rev Lett* **2003**, *91*, 233901.

[26] B. Ren, G. Picardi, B. Pettinger, *Review of Scientific Instruments* **2004**, *75*, 837-841.

[27] K. Karrai, R. D. Grober, *Applied Physics Letters* **1995**, *66*, 1842-1844.

[28] a) M. Hegner, P. Wagner, G. Semenza, *Surface Science* **1993**, *291*, 39-46; b) N. Vogel, J. Zieleniecki, I. Koper, *Nanoscale* **2012**, *4*, 3820-3832; c) W. Chen, S. Zhang, M. Kang, W. Liu, Z. Ou, Y. Li, Y. Zhang, Z. Guan, H. Xu, *Light Sci Appl* **2018**, *7*, 56.

[29] M. P. Seah, W. A. Dench, *Surface and Interface Analysis* **2004**, *1*, 2-11.

[30] J. J. Yeh, I. Lindau, *Atomic Data and Nuclear Data Tables* **1985**, *32*, 1-155.

[31] H. Peisert, J. Uihlein, F. Petraki, T. Chassé, *Journal of Electron Spectroscopy and Related Phenomena* **2015**, *204*, 49-60.

[32] a) T. D. Kuhne, M. Iannuzzi, M. Del Ben, V. V. Rybkin, P. Seewald, F. Stein, T. Laino, R. Z. Khaliullin, O. Schutt, F. Schiffmann, D. Golze, J. Wilhelm, S. Chulkov, M. H. Bani-Hashemian, V. Weber, U. Borstnik, M. Taillefumier, A. S. Jakobovits, A. Lazzaro, H. Pabst, T. Muller, R. Schade, M. Guidon, S. Andermatt, N. Holmberg, G. K. Schenter, A. Hehn, A. Bussy, F. Belleflamme, G. Tabacchi, A. Gloss, M. Lass, I. Bethune, C. J. Mundy, C. Plessl, M. Watkins, J. VandeVondele, M. Krack, J. Hutter, *J Chem Phys* **2020**, *152*, 194103; b) J. VandeVondele, M. Krack, F. Mohamed, M. Parrinello, T. Chassaing, J. Hutter, *Computer Physics Communications* **2005**, *167*, 103-128.

[33] J. P. Perdew, K. Burke, M. Ernzerhof, *Phys Rev Lett* **1996**, *77*, 3865-3868.

[34] a) S. Grimme, S. Ehrlich, L. Goerigk, *J Comput Chem* **2011**, *32*, 1456-1465; b) S. Grimme, J. Antony, S. Ehrlich, H. Krieg, *J Chem Phys* **2010**, *132*, 154104.

[35] a) F. Hanke, J. Björk, *Physical Review B* **2013**, *87*; b) D. A. Egger, Z. F. Liu, J. B. Neaton, L. Kronik, *Nano Lett* **2015**, *15*, 2448-2455.

[36] R. W. G. Wyckoff, R. W. Wyckoff, *Crystal structures, Vol. Vol. 1*, Interscience publishers, New York, **1963**.

[37] M. J. Frisch, G. W. Trucks, H. B. Schlegel, G. E. Scuseria, M. A. Robb, J. R. Cheeseman, G. Scalmani, V. Barone, G. A. Petersson, H. Nakatsuji, X. Li, M. Caricato, A. V. Marenich, J. Bloino, B. G. Janesko, R. Gomperts, B. Mennucci, H. P. Hratchian, J. V. Ortiz, A. F. Izmaylov, J. L. Sonnenberg, Williams, F. Ding, F. Lipparini, F. Egidi, J. Goings, B. Peng, A. Petrone, T. Henderson, D. Ranasinghe, V. G. Zakrzewski, J. Gao, N. Rega, G. Zheng, W. Liang, M. Hada, M. Ehara, K. Toyota, R. Fukuda, J. Hasegawa, M. Ishida, T. Nakajima, Y. Honda, O. Kitao, H. Nakai, T. Vreven, K. Throssell, J. A. Montgomery Jr., J. E. Peralta, F. Ogliaro, M. J. Bearpark, J. J. Heyd, E. N. Brothers, K. N. Kudin, V. N.

Staroverov, T. A. Keith, R. Kobayashi, J. Normand, K. Raghavachari, A. P. Rendell, J. C. Burant, S. S. Iyengar, J. Tomasi, M. Cossi, J. M. Millam, M. Klene, C. Adamo, R. Cammi, J. W. Ochterski, R. L. Martin, K. Morokuma, O. Farkas, J. B. Foresman, D. J. Fox, Gaussian 16 Revision C.01, **2016**, Gaussian Inc. Wallingford, CT

[38] C. I. Bayly, P. Cieplak, W. Cornell, P. A. Kollman, *The Journal of Physical Chemistry* **2002**, *97*, 10269-10280.

[39] F. Schneider, L. Lang, L. Wang, J. Gierschner, A. J. Meixner, M. Fleischer, D. Zhang, *The Journal of Physical Chemistry C* **2024**, *128*, 18807-18819.

[40] S. M. Song, F.; Li, X.; Yin, X.; Li, H., *Digest Journal of Nanomaterials and Biostructures* **2018**, *13*, 7-12.

[41] a) K. Q. Lin, J. Yi, J. H. Zhong, S. Hu, B. J. Liu, J. Y. Liu, C. Zong, Z. C. Lei, X. Wang, J. Aizpurua, R. Esteban, B. Ren, *Nat Commun* **2017**, *8*, 14891; b) S. Jäger, A. M. Kern, M. Hentschel, R. Jäger, K. Braun, D. Zhang, H. Giessen, A. J. Meixner, *Nano Lett* **2013**, *13*, 3566-3570.

[42] a) W. Yu, *Electromagnetic Simulation Techniques Based on the FDTD Method*, Wiley & Sons, **2009**; b) D. B. Y. Ge, Y.B., *Finite-difference time-domain method for electromagnetic waves (in chinese)*, Xi'an: Xidian University Press, **2011**.

[43] a) P. B. Johnson, R. W. Christy, *Physical Review B* **1972**, *6*, 4370-4379; b) T. V. Basova, N. S. Mikhaleva, A. K. Hassan, V. G. Kiselev, *Sensors and Actuators B: Chemical* **2016**, *227*, 634-642.

[44] Y. Cao, Y. Feng, Y. Cheng, L. Meng, M. Sun, *Applied Physics Letters* **2023**, *122*.

[45] a) C. Höppener, J. Aizpurua, H. Chen, S. Gräfe, A. Jorio, S. Kupfer, Z. Zhang, V. Deckert, *Nature Reviews Methods Primers* **2024**, *4*; b) Y. Zhang, B. Yang, A. Ghafoor, Y. Zhang, Y. F. Zhang, R. P. Wang, J. L. Yang, Y. Luo, Z. C. Dong, J. G. Hou, *Natl Sci Rev* **2019**, *6*, 1169-1175.

[46] W. Zhu, D. Wang, K. B. Crozier, *Nano Lett* **2012**, *12*, 6235-6243.
